# The ULK complex–LRRK1 axis regulates Parkin-mediated mitophagy via Rab7 Ser-72 phosphorylation

**DOI:** 10.1242/jcs.260395

**Published:** 2022-12-07

**Authors:** Keitaro Fujita, Shin Kedashiro, Takuya Yagi, Naoki Hisamoto, Kunihiro Matsumoto, Hiroshi Hanafusa

**Affiliations:** Division of Biological Science, Graduate School of Science, Nagoya University, Nagoya 464-8602, Japan

**Keywords:** LRRK1, Rab7, Mitophagy

## Abstract

Mitophagy, a type of selective autophagy, specifically targets damaged mitochondria. The ULK complex regulates Parkin-mediated mitophagy, but the mechanism through which the ULK complex initiates mitophagosome formation remains unknown. The Rab7 GTPase (herein referring to Rab7a) is a key initiator of mitophagosome formation, and Ser-72 phosphorylation of Rab7 is important for this process. We have previously identified LRRK1 as a protein kinase responsible for Rab7 Ser-72 phosphorylation. In this study, we investigated the role of LRRK1 in mitophagy. We showed that LRRK1 functions downstream of ULK1 and ULK2 in Parkin-mediated mitophagy. Furthermore, we demonstrated that ectopic targeting of active LRRK1 to mitochondria is sufficient to induce the Ser-72 phosphorylation of Rab7, circumventing the requirement for ATG13, a component of the ULK complex. Thus, the ULK complex recruits LRRK1 to mitochondria by interacting with ATG13 to initiate mitophagosome formation. This study highlights the crucial role of the ULK complex–LRRK1 axis in the regulation of Parkin-mediated mitophagy.

## Teaser

**Summary:** LRRK1 is recruited to damaged mitochondria by interacting with ATG13, a component of the ULK complex, during Parkin-mediated mitophagy and initiates mitophagosome formation via Rab7 Ser-72 phosphorylation.

## INTRODUCTION

Autophagy is an evolutionarily conserved cellular process that removes unnecessary or dysfunctional cellular components (Mizushima et al., 2011). Mitophagy is a selective form of autophagy that eliminates damaged mitochondria and, therefore, plays a key role in mitochondrial quality control (Palikaras et al., 2018; Pickles et al., 2018; Killackey et al., 2020). Damaged mitochondria are first tagged by ubiquitin (Ub), and autophagy receptors then bind the ubiquitinated cargo through their Ub-binding domain (Stolz et al., 2014; Gatica et al., 2018). Autophagy-related (ATG) proteins assemble to engulf the cargo into a novel, double-membraned organelle, called the autophagosome, and deliver it to the lysosome for content degradation (Mizushima et al., 2011; Bento et al., 2016; Nakatogawa, 2020). Defective mitochondria cause oxidative stress, which can compromise the health of the entire mitochondrial network. Several lines of evidence suggest that mitochondrial dysfunction is strongly associated with the pathogenesis of neurodegenerative disorders, such as Parkinson's disease (PD) (Pickrell and Youle, 2015; Bose and Beal, 2016; McWilliams and Muqit, 2017; Killackey et al., 2020).

The molecular mechanisms of mitophagy have been extensively studied, resulting in the discovery of several important signaling pathways. The most well-understood form of mitophagy is orchestrated by the mitochondrial kinase PTEN-induced kinase 1 (PINK1) and the E3-Ub ligase Parkin, which are encoded by two genes that are mutated in familial PD (Pickrell and Youle, 2015; McWilliams and Muqit, 2017). Genetic studies have established that PINK1 acts upstream of Parkin (Clark et al., 2006; Park et al., 2006; Yang et al., 2006). PINK1 is normally imported into the mitochondria and rapidly degraded. However, when the mitochondria are damaged, PINK1 is stabilized on the outer mitochondrial membrane (OMM) (Matsuda et al., 2010; Narendra et al., 2010), where it phosphorylates both Parkin and Ub, recruiting and activating Parkin (Narendra et al., 2008, 2010; Geisler et al., 2010; Matsuda et al., 2010; Kondapalli et al., 2012; Shiba-Fukushima et al., 2012; Kane et al., 2014; Kazlauskaite et al., 2014; Koyano et al., 2014). Subsequently, the Parkin-mediated ubiquitylation of OMM proteins in combination with PINK1-mediated Ub phosphorylation triggers the recruitment of autophagy receptors, such as NDP52 (also known as CALCOCO2) and optineurin (OPTN). These receptors connect Ub-tagged mitochondria to the upstream autophagy machinery, including LC3 family proteins (also known as MAP1LC3 proteins), to initiate mitophagosome biogenesis (Birgisdottir et al., 2013; Lazarou et al., 2015; Wang et al., 2020). Consequently, these receptors facilitate the selective engulfment of damaged mitochondria into the mitophagosome. The ability of these Ub-binding receptors to promote efficient mitophagy requires TANK-binding kinase 1 (TBK1), which phosphorylates the receptor upon binding to the Ub chain (Heo et al., 2015; Matsumoto et al., 2015; Richter et al., 2016). Emerging evidence suggests that the ULK multiprotein complex is involved in mitophagy. The ULK complex consists of the ULK1 or ULK2 kinases, FIP200 (also known as RB1CC1), ATG13 and ATG101 (Hurley and Young, 2017). The mitophagy receptor NDP52 forms a complex with FIP200. NDP52 and TBK1 then cooperate to recruit the ULK complex into damaged mitochondria, inducing mitophagosome formation (Vargas et al., 2019). Although the ULK complex plays a critical role in the mitophagosome formation, only a few downstream players of ULK have been identified. The mechanisms underlying the role of ULK in mitophagy remain to be elucidated.

Recent studies have identified Rab7a guanosine triphosphatase (GTPase) (hereafter referred to as Rab7) as a key component in the initiation of mitophagosome formation in Parkin-mediated mitophagy (Jimenez-Orgaz et al., 2018; Yamano et al., 2018). The Rab family functions as regulators of membrane trafficking by switching between a membrane-associated GTP-bound active form and a cytosolic GDP-bound inactive form (Stenmark, 2009). Rab7 is recruited to damaged mitochondria during the early stages of Parkin-mediated mitophagy and induces mitophagosome formation around damaged mitochondria by targeting ATG9 (herein referring to ATG9A)-containing vesicles, which serve as a membrane source for mitophagosome formation (Yamamoto et al., 2012; Jimenez-Orgaz et al., 2018; Yamano et al., 2018). Interestingly, phosphorylation of Rab7 Ser-72 appears to be required for ATG9 recruitment (Heo et al., 2018). TBK1 directly phosphorylates the Ser-72 residue in Rab7 *in vitro*. Ser-72 is located in the switch-II domain, which is involved in both GDP–GTP exchange and interactions with other proteins (Pfeffer, 2005). Residues that are phosphorylated are present at the corresponding positions in most Rab family members. LRRK2 protein kinase, which also undergoes mutation in PD, phosphorylates similar positions on a distinct set of Rab proteins to control interactions with its regulators (Steger et al., 2016, 2017). For example, the LRRK2 phosphorylation of Thr-73 in Rab10, corresponding to Ser-72 in Rab7, participates in mitophagy (Wauters et al., 2019).

We recently identified LRRK1 as a kinase responsible for the phosphorylation of Rab7 at Ser-72 (Hanafusa et al., 2019). The LRRK1-mediated phosphorylation of Rab7 Ser-72 links the trafficking of the epidermal growth factor receptor (EGFR)-containing endosome to its effector RILP. LRRK1 is related to LRRK2, and both belong to the ROCO family of proteins (Bosgraaf and Van Haastert, 2003). Several recent reports have demonstrated that LRRK1 and LRRK2 regulate autophagy (Toyofuku et al., 2015; Bonello et al., 2019; Wauters et al., 2019). Therefore, we studied the role of LRRK1 in the process of Parkin-mediated mitophagy. Here, we identified a novel signaling pathway in which the ULK complex–LRRK1 axis regulates Parkin-mediated mitophagy. This study reveals that LRRK1 positively regulates Parkin-mediated mitophagy and provides a striking example of signaling pathway involvement in membrane trafficking to regulate cell function.

## RESULTS

### LRRK1 is required for Parkin-mediated mitophagy

**Fig. 1.**
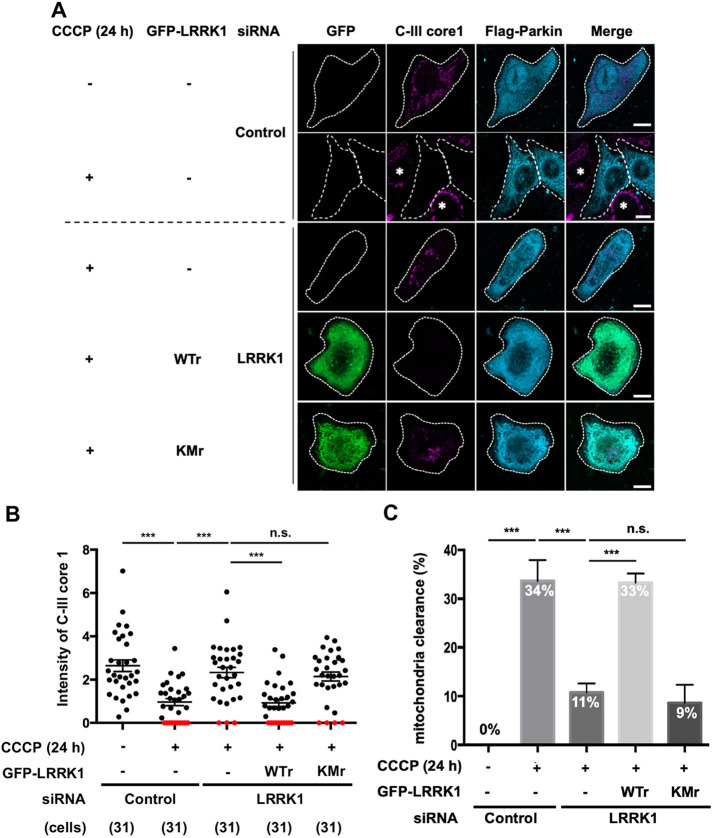
**LRRK1 is involved in Parkin-mediated mitophagy.** (A) Effect of LRRK1 depletion on the elimination of depolarized mitochondria. U2OS cells treated with either control siRNA or LRRK1 siRNA were transfected with Flag–Parkin and siRNA-resistant wild-type GFP–LRRK1 (WTr) or kinase-negative GFP–LRRK1 (KMr), as indicated. After 24 h of treatment with or without CCCP (10 µM), the cells were immunostained with antibodies against C-III core 1 (magenta) for the IMM and mitochondrial matrix, and Flag (cyan) for Parkin. White dotted lines indicate Flag–Parkin-expressing cells. Asterisks indicate cells that did not express Flag–Parkin. Scale bars: 10 µm. (B) Quantification of mitochondrial mass. Data were plotted as the fluorescence intensity of C-III core 1 in Flag–Parkin-expressing cells after background subtraction. The number of cells examined is indicated. Red circles indicate cells with mitochondria clearance. A typical example of an experiment conducted three times is shown. (C) Quantification of mitochondrial clearance. Data represent the percentage of Flag–Parkin-expressing cells without mitochondria per total Flag–Parkin-expressing cells (*n*=3; >30 cells counted per condition). Error bars in B and C represent s.d. ***P<0.001, n.s. not significant (Dunnett's multiple-comparison test).

Parkin is specifically recruited to the mitochondria in response to mitochondrial depolarization induced by the proton uncoupler carbonyl cyanide 3-chlorophenylhydrazone (CCCP) (Narendra et al., 2008). Through this process, damaged mitochondria are eliminated via mitophagy. To determine the role of LRRK1 in Parkin-mediated mitophagy, we evaluated the effect of LRRK1 depletion on the clearance of depolarized mitochondria in human U2OS cells lacking endogenous Parkin expression (Lei et al., 2018). The U2OS cells were transiently transfected with Flag-tagged Parkin. Then, mitophagy was measured by immunostaining with antibodies against Flag and the core 1 subunit complex III (C-III) of the inner mitochondrial membrane (IMM) protein as a mitochondrial marker (Yoshii et al., 2011; Lazarou et al., 2015). As previously reported (Narendra et al., 2008), CCCP stimulation markedly reduced mitochondrial mass in Flag–Parkin-expressing cells (Fig. 1A,B), and ∼30% of these cells completely lost mitochondrial staining 24 h after CCCP treatment (Fig. 1A,C). Mitochondrial clearance was not observed in the absence of Parkin (Fig. 1A), confirming that depolarized mitochondria are eliminated by Parkin-dependent mitophagy. When U2OS cells expressing Flag–Parkin were treated with LRRK1 siRNA (Fig. S1A), the percentage of cells lacking mitochondrial staining after 24 h of CCCP treatment was significantly reduced (Fig. 1A–C). These results indicate that LRRK1 is necessary for Parkin-mediated mitophagy induced by CCCP.

To investigate whether LRRK1 kinase activity is required for the removal of depolarized mitochondria, we conducted rescue experiments using an LRRK1 kinase-negative mutant [LRRK1(KM)], in which Lys-1243 in the LRRK1 kinase domain was replaced with methionine (Hanafusa et al., 2011). In LRRK1-depleted cells, the expression of siRNA-resistant wild-type GFP-tagged LRRK1, but not siRNA-resistant GFP–LRRK1(KM), restored the deficiency in CCCP-induced mitochondrial clearance (Fig. 1A–C). These results indicate that LRRK1 kinase activity is necessary for Parkin-mediated mitophagy. Furthermore, we obtained comparable results with the ATP synthase inhibitor oligomycin and the complex III inhibitor antimycin A (O/A) as signals (Fig. S2A–C) (Yoshii et al., 2011; Lazarou et al., 2015).

### LRRK1 phosphorylates Rab7 Ser-72 on depolarized mitochondria

**Fig. 2.**
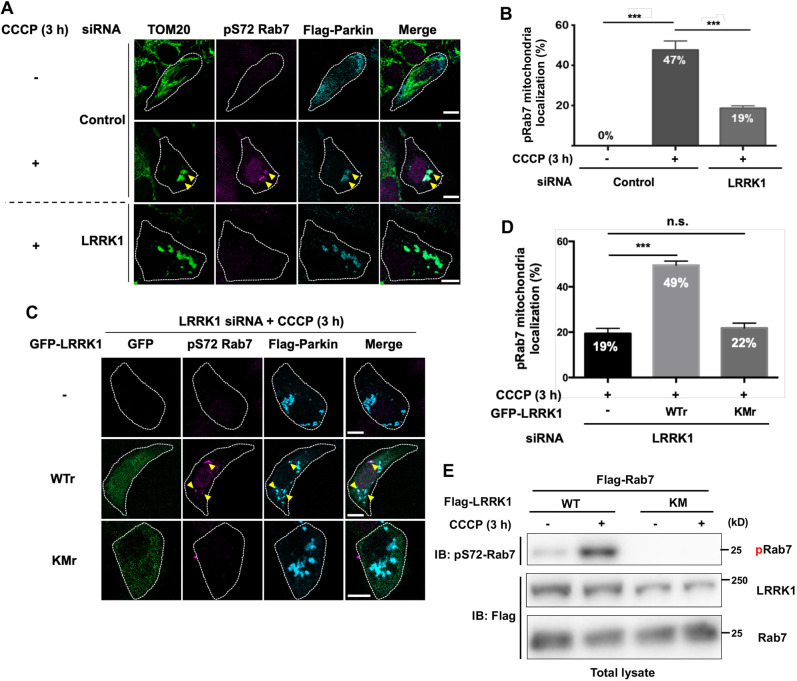
**LRRK1 is required for Rab7 Ser-72 phosphorylation on depolarized mitochondria.** (A,C) Effect of LRRK1 depletion on mitochondrial Rab7 Ser-72 phosphorylation. U2OS cells treated with either control siRNA or LRRK1 siRNA were transfected with Flag–Parkin and siRNA-resistant wild-type GFP–LRRK1 (WTr) or kinase-negative GFP-LRRK1 (KMr), as indicated. After 3 h of treatment with or without CCCP (10 µM), the cells were immunostained with antibodies against TOM20 (green) for mitochondria, pS72-Rab7 (magenta), and Flag (cyan) for Parkin. White dotted lines indicate Flag–Parkin-expressing cells. Yellow arrowheads indicate the pS72-Rab7 signal on mitochondria decorated with Flag–Parkin. Scale bars: 10 µm. (B,D) Quantification of the colocalization of pSer-72 Rab7 and Flag–Parkin. Data represent the percentage of Flag–Parkin-expressing cells with pS72 Rab7 signals on structures decorated with Flag–Parkin per total Flag–Parkin-expressing cells [*n*=3; >70 cells counted per condition (B), *n*=3; >30 cells counted per condition (D)]. Error bars represent s.d. ***P<0.001, n.s., not significant (Dunnett's multiple-comparison test). (E) Activation of LRRK1 by CCCP. HEK293 cells were co-transfected with Flag–LRRK1 (WT or KM) and Flag–Rab7 and treated with or without CCCP for 3 h. Cell lysates were immunoblotted (IB) with antibodies as indicated. Blot shown is representative of three repeats.

Rab7 plays a significant role in the early stages of Parkin-mediated mitophagy, especially in mitophagosome formation, by targeting ATG9-containing vesicles around damaged mitochondria (Jimenez-Orgaz et al., 2018; Yamano et al., 2018). Ser-72 phosphorylation in Rab7 appears to be crucial for this process (Heo et al., 2018). We therefore analyzed the distribution of endogenous Rab7 phosphorylated at Ser-72 (pS72-Rab7) in mitophagy using an antibody specific for pS72-Rab7. As previously observed (Narendra et al., 2008), treatment of U2OS cells expressing Flag–Parkin with CCCP or O/A for 3 h resulted in a rapid translocation of Flag–Parkin from the cytosol to the mitochondria (Fig. 2A; Fig. S3A). The mitochondrial network collapsed around the perinuclear region, and Flag–Parkin was localized throughout the depolarized mitochondria (Fig. 2A; Fig. S3A). We observed a pattern of pS72-Rab7 on the punctate structure colocalized with Flag–Parkin (Fig. 2A,B; Fig. S3A,B). This indicates that Rab7 Ser-72 is phosphorylated on depolarized mitochondria during Parkin-mediated mitophagy.

We recently reported that LRRK1 phosphorylates Ser-72 in GTP-bound Rab7 at the endosomal membrane (Hanafusa et al., 2019). Then, we examined whether LRRK1 participates in Ser-72 phosphorylation in Parkin-mediated mitophagy. In Flag–Parkin-expressing cells, the depletion of LRRK1 with siRNA and treatment with CCCP or O/A significantly reduced the pS72-Rab7 signal on damaged mitochondria (Fig. 2A,B; Fig. S3A,B). By contrast, LRRK1 depletion had no effect on the CCCP- or O/A-induced mitochondrial aggregation or Parkin translocation to the mitochondria (Fig. 2A and Fig. S3A), suggesting that LRRK1 is dispensable for the recruitment of Parkin to damaged mitochondria. Consistent with the fact that LRRK1 phosphorylates Rab7 Ser-72 (Hanafusa et al., 2019), we found that the expression of siRNA-resistant LRRK1(KM) in LRRK1-depleted cells was unable to restore the loss of the pS72-Rab7 signal on the mitochondria (Fig. 2C,D). Given that Rab7 is recruited to the damaged mitochondria after depolarization (Jimenez-Orgaz et al., 2018; Yamano et al., 2018), these results indicate that LRRK1 phosphorylates Rab7 Ser-72 on the damaged mitochondria during Parkin-mediated mitophagy.

We next examined whether LRRK1 kinase is activated in Parkin-mediated mitophagy. We monitored the LRRK1 kinase activity in cell extracts by assessing pS72-Rab7 in HEK293 cells expressing endogenous Parkin. Briefly, the cells were co-transfected with Flag–LRRK1 and Flag–Rab7, treated with CCCP for 3 h, and immunoblotted with the anti-pS72-Rab7 antibody. We found that co-transfection with Flag–LRRK1, but not with LRRK1(KM), induced Rab7 phosphorylation after CCCP treatment (Fig. 2E). Thus, CCCP treatment activates LRRK1, which then phosphorylates Ser-72 in Rab7 in response.

### Relationship between LRRK1 and ULK kinase in CCCP-induced Rab7 Ser-72 phosphorylation

**Fig. 3.**
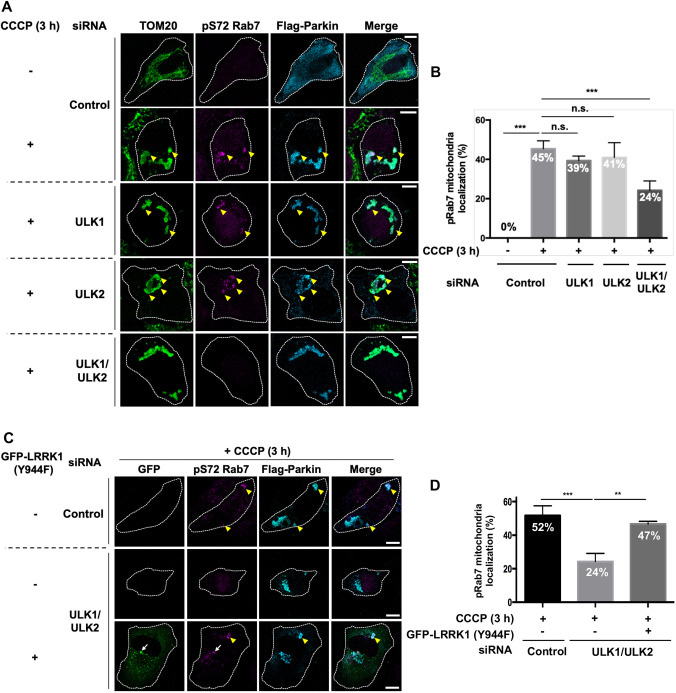
**LRRK1 regulates CCCP-induced Rab7 Ser-72 phosphorylation downstream of ULK1 and ULK2.** (A,C) Effect of ULK1 and ULK2 double-depletion (ULK1/2) on the phosphorylation of Rab7 Ser-72. U2OS cells treated with control siRNA, ULK1 siRNA, ULK2 siRNA, or both ULK1/ULK2 siRNAs were transfected with Flag–Parkin and GFP–LRRK1(Y944F), as indicated. After 3 h of treatment with or without CCCP (10 µM), the cells were immunostained with the antibodies against TOM20 (green) for mitochondria, pS72-Rab7 (magenta), and Flag (cyan) for Parkin. White dotted lines indicate Flag–Parkin-expressing cells. Yellow arrowheads and white arrows indicate the pS72-Rab7 signals on mitochondria decorated with Flag–Parkin and endosomes decorated with GFP–LRRK1(Y944F), respectively. Scale bars: 10 µm. (B,D) Quantification of the colocalization of pSer-72 Rab7 and Flag–Parkin. Data represent the percentage of Flag–Parkin-expressing cells with pS72 Rab7 signals on structures decorated with Flag–Parkin per total Flag–Parkin-expressing cells (*n*=3; >30 cells counted per condition). Error bars represent s.d. **P<0.01; ***P<0.001, n.s., not significant (Dunnett's multiple-comparison test).

The ULK kinase in the ULK multiprotein complex is an upstream-acting component of the macroautophagy machinery (Hurley and Young, 2017). A recent study has demonstrated that the recruitment and activation of ULK in the mitochondria are pivotal steps in Parkin-mediated mitophagy (Vargas et al., 2019). Therefore, we investigated the relationship between LRRK1 and ULK in the regulation of Rab7 Ser-72 phosphorylation in Parkin-mediated mitophagy. First, we analyzed the effect of ULK depletion on CCCP-induced mitochondrial Ser-72 phosphorylation in Rab7. In Flag–Parkin-expressing U2OS cells treated with ULK1 or ULK2 siRNA (Fig. S1B), punctate pS72-Rab7 signals persisted in CCCP-induced depolarized mitochondria (Fig. 3A,B). By contrast, when ULK1 and ULK2 were depleted simultaneously, these signals were significantly reduced (Fig. 3A,B). Thus, ULK1 and ULK2 redundantly regulate Rab7 Ser-72 phosphorylation on damaged mitochondria in response to CCCP treatment.

To elucidate the relationship between ULK1, ULK2 and LRRK1, we examined the effect of the constitutively active LRRK1 mutant LRRK1(Y944F) (Ishikawa et al., 2012) on the phenotype caused by ULK1 and/or ULK2 siRNAs. We found that the expression of LRRK1(Y944F) in ULK1 and ULK2 double-depleted cells suppressed the loss of Ser-72 phosphorylation in Rab7 on the damaged mitochondria (Fig. 3C,D). Note that the punctate GFP–LRRK1(Y944F) signal colocalized with pS72-Rab7 but not with Flag–Parkin (Fig. 3C) because LRRK1(Y944F) accumulates in the endosome and phosphorylates Rab7 Ser-72 (Hanafusa et al., 2019). These results indicate that LRRK1 acts downstream of ULK1 and ULK2 to regulate CCCP-induced Rab7 Ser-72 phosphorylation.

### Relationship between LRRK1 and ATG13 in Parkin-mediated mitophagy

Next, we examined the relationship between LRRK1 and the ULK complex in Parkin-mediated mitophagy. Considering that the ULK complex is composed of the ULK1 or ULK2 kinase, FIP200, ATG13 and ATG101 (Hurley and Young, 2017), we examined whether LRRK1 interacts with these components. GFP–LRRK1 was co-transfected into HEK293 cells along with Flag–ATG13, Flag–ATG101, Flag–FIP200 or HA–ULK1. We found that LRRK1 bound to ATG13 and ATG101 (Fig. S4A), but not to ULK1 (Fig. S4B). However, the interaction between LRRK1 and FIP200 remained unclear because of the low expression level of FIP200 (Fig. S4A).

**Fig. 4.**
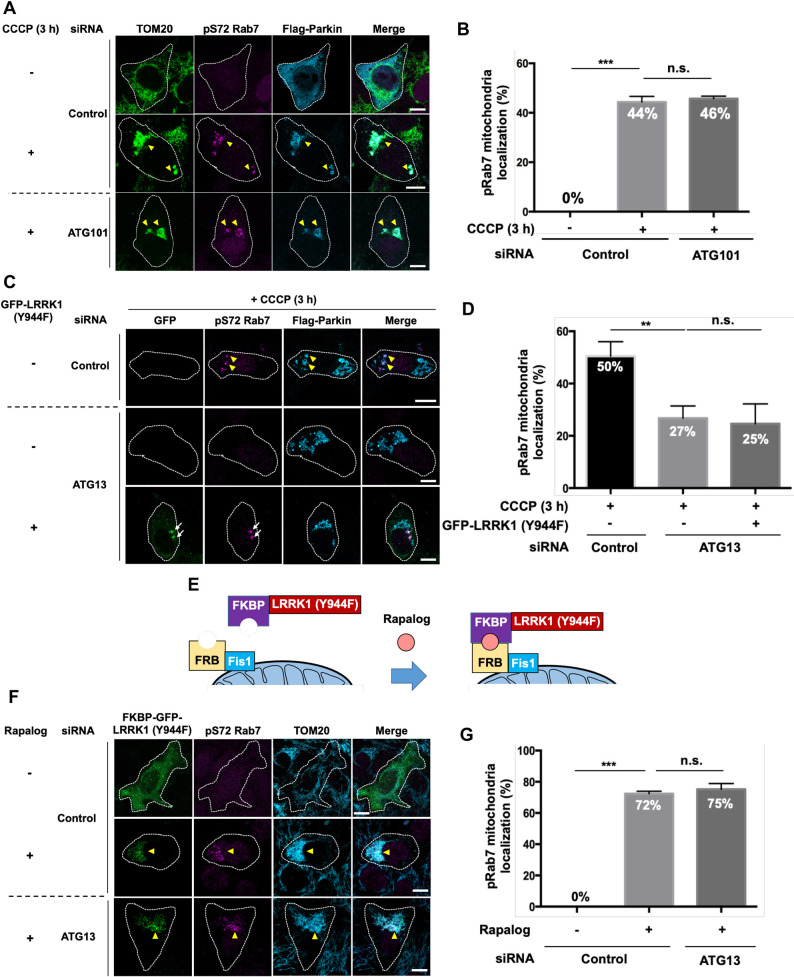
**Effects of ATG101 or ATG13 depletion on CCCP-induced Rab7 Ser-72 phosphorylation.** (A) Effect of ATG101 depletion on mitochondrial Rab7 Ser-72 phosphorylation. U2OS cells treated with either control siRNA or ATG101 siRNA were transfected with Flag–Parkin, as indicated. After 3 h of treatment with or without CCCP (10 μM), the cells were immunostained with antibodies against TOM20 (green) for mitochondria, pS72-Rab7 (magenta), and Flag (cyan) for Parkin. White dotted lines indicate Flag-Parkin-expressing cells. Yellow arrowheads indicate the pS72-Rab7 signal on mitochondria decorated with Flag-Parkin. Scale bars: 10 µm. (B,D) Quantification of the colocalization of pSer-72 Rab7 and Flag–Parkin. Data represent the percentage of Flag–Parkin-expressing cells with pS72 Rab7 signals on structures decorated with Flag–Parkin per total Flag–Parkin-expressing cells (*n*=3; >30 cells counted per condition). The error bars represent s.d. **P<0.01, ***P<0.001, n.s., not significant (Dunnett's multiple-comparison test). (C) Effect of ATG13 depletion on mitochondrial Rab7 Ser-72 phosphorylation. U2OS cells treated with control siRNA or ATG13 siRNA were transfected with Flag–Parkin and empty vector or GFP–LRRK1(Y944F), as indicated. After 3 h of CCCP treatment (10 µM), the cells were immunostained with antibodies against pS72-Rab7 (magenta) and Flag (cyan) for Parkin. White dotted lines indicate GFP–LRRK1(Y944F)-expressing cells. Yellow arrowheads and white arrows indicate pS72-Rab7 signals on the mitochondria decorated with Flag–Parkin and endosomes decorated with GFP-LRRK1(Y944F), respectively. Scale bars: 10 µm. (E) Schematic of a chemically induced dimerization assay to control mitochondrial localization of LRRK1(Y944F). (F) Effect of artificially targeting LRRK1(Y944F) to the mitochondria on the phosphorylation of Rab7 Ser-72. U2OS cells treated with control siRNA or ATG13 siRNA were co-transfected with FKBP–GFP–LRRK1(Y944F) and FRB–Fis1. Cells were treated with or without rapalog (0.5 µM) for 24 h and immunostained with antibodies against pS72-Rab7 (magenta) and TOM20 (cyan) for mitochondria. White dotted lines indicate GFP–LRRK1(Y944F)-expressing cells. Yellow arrowheads indicate the pS72-Rab7 signal on mitochondria decorated with GFP–LRRK1(Y944F). Scale bars: 10 µm. (G) Quantification of the colocalization of pSer-72 Rab7 and TOM20. Data represent the percentage of GFP–LRRK1(Y944F)-expressing cells with pS72 Rab7 signals on mitochondria decorated with GFP–LRRK1(Y944F) per total GFP–LRRK1(Y944F)-expressing cells (*n*=3; >30 cells counted per condition). The error bars represent s.d. ***P<0.001, n.s., not significant (Dunnett's multiple-comparison test).

We next investigated whether ATG101 and ATG13 are required for Rab7 Ser-72 phosphorylation on depolarized mitochondria. ATG101 knockdown with siRNA in Flag–Parkin-expressing U2OS cells did not affect the Ser-72 phosphorylation of Rab7 induced by CCCP (Fig. 4A,B; Fig. S1C). In contrast, the knockdown of ATG13 reduced CCCP-induced mitochondrial Rab7 phosphorylation (Fig. 4C,D; Fig. S1D). Considering that LRRK1 functions downstream of ULK1 and ULK2 in Parkin-mediated mitophagy, we hypothesized that the constitutively active LRRK1(Y944F) mutation would suppress the loss of Rab7 phosphorylation by ATG13 knockdown. However, compared with ULK1 and ULK2 double-knockdown cells, LRRK1(Y944F) expression did not suppress the loss of Rab7 Ser-72 phosphorylation observed in ATG13-depleted cells (Fig. 4C,D).

Why does LRRK1(Y944F) suppress ULK1 and ULK2 depletion but not ATG13 knockdown? The ULK complex is recruited to the damaged mitochondria, leading to ULK kinase activation (Vargas et al., 2019). Therefore, the ULK complex-dependent recruitment of LRRK1 to mitochondria might be necessary for Rab7 Ser-72 phosphorylation on the depolarized mitochondria. Although we could not detect GFP–LRRK1 localization to the mitochondria upon mitophagy activation (Fig. 2C), we examined whether artificially targeting LRRK1(Y944F) to the mitochondria would suppress ATG13 siRNA-induced deficiency. To test this scenario, we used a chemically induced dimerization (CID) assay to recruit LRRK1(Y944F) directly to the mitochondria. The CID assay utilizes rapamycin analog (rapalog)-dependent dimerization of the FK506-binding protein (FKBP) and FKBP12-rapamycin-binding (FRB) domain (Clackson et al., 1998; Vargas et al., 2019). The FKBP and FRB domains were fused to GFP–LRRK1(Y944F) and the OMM protein Fis1, respectively (Fig. 4E). FKBP–GFP–LRRK1(Y944F) and FRB–Fis1 were co-expressed in U2OS cells. Rapalog treatment efficiently induced the translocation of GFP–LRRK1(Y944F) to the mitochondria, inducing the pS72-Rab7 signal on LRRK1-decorated mitochondria (Fig. 4F,G). The addition of rapalog to cells expressing only FKBP–GFP–LRRK1(Y944F) did not induce LRRK1(Y944F) translocation to the mitochondria or Rab7 Ser-72 phosphorylation (Fig. S5). We also found that the tethering of LRRK1(Y944F) to the mitochondria triggered Rab7 Ser-72 phosphorylation in ATG13 siRNA-treated U2OS cells (Fig. 4F,G). These results suggest that ATG13 mediates mitochondrial localization of LRRK1 during Parkin-dependent mitophagy.

### LRRK1 interacts with ATG13 in response to mitophagy activation

**Fig. 5.**
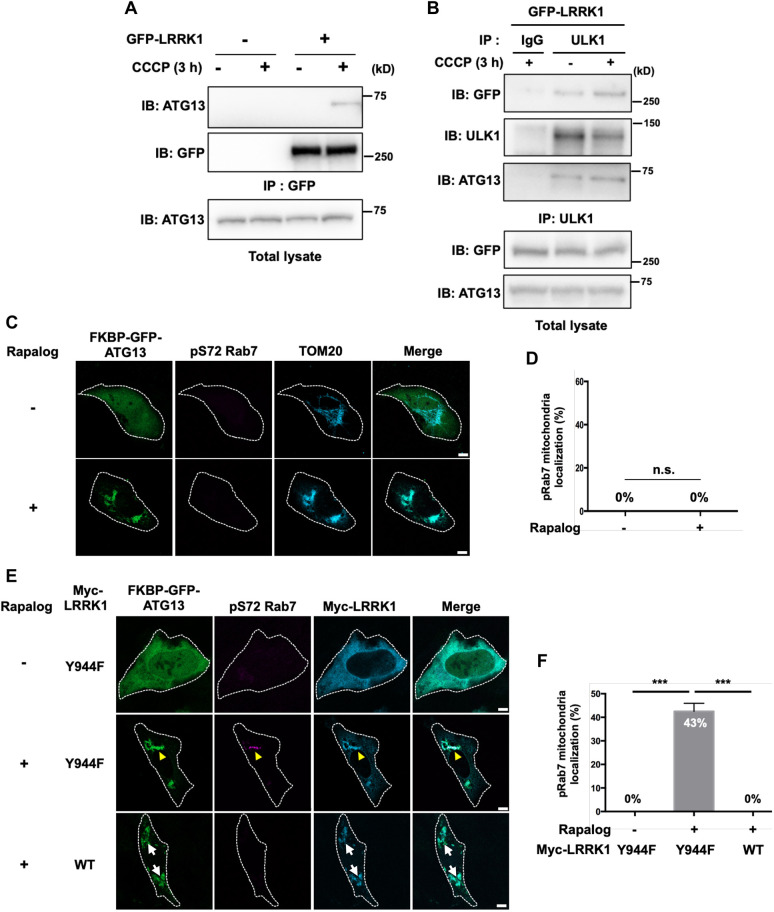
**Effect of artificially targeting ATG13 to the mitochondria on the phosphorylation of Rab7 Ser-72.** (A) Interaction between LRRK1 and ATG13. HEK293 cells were transfected with GFP–LRRK1. Cells were treated with or without CCCP for 3 h. Complex formation was detected by immunoprecipitation (IP) with anti-GFP antibodies, followed by immunoblotting (IB) with antibodies, as indicated. (B) Interaction between LRRK1 and ULK1. HEK293 cells were transfected with GFP–LRRK1. Cells were treated with or without CCCP for 3 h. Complex formation was detected by immunoprecipitation (IP) with anti-ULK1 antibodies, followed by immunoblotting (IB) with antibodies as indicated. Blots shown are representative of two repeats. (C,E) U2OS cells were co-transfected with FKBP–GFP–ATG13 and FRB–Fis1 in the presence (E) or absence (C) of Myc–LRRK1(Y944F or wild-type, WT), as indicated. Cells were treated with or without rapalog (0.5 µM) for 24 h and immunostained with the following antibodies against pS72-Rab7 (magenta), Myc, and TOM20 (cyan) for mitochondria. Yellow arrowheads indicate the pS72-Rab7 signal on the mitochondria decorated with GFP–ATG13. White arrows indicate the Myc–LRRK1 signal on the mitochondria decorated with GFP–ATG13. Scale bars: 10 µm. (D,F) Quantification of the colocalization of pSer-72 Rab7, TOM20 and GFP-ATG13. Data represent the percentage of GFP–ATG13-expressing cells with pS72 Rab7 signals on mitochondria decorated with GFP–ATG13 per total GFP-ATG13-expressing cells (*n*=3; >30 cells counted per condition). The error bars represent s.d. ***P<0.001, n.s., not significant (Welch's *t*-test).

The above-mentioned results suggest that ATG13 interacts with and recruits LRRK1 to the mitochondria during Parkin-mediated mitophagy. We also found that overexpressed LRRK1 interacted with endogenous ATG13 in a CCCP or O/A stimulation-dependent manner (Fig. 5A; Fig. S4C). Similarly, the interaction between overexpressed LRRK1 and endogenous ULK1 was enhanced after CCCP stimulation (Fig. 5B). When LRRK1 and ULK1 were overexpressed, no interaction was observed (Fig. S4B). This finding suggests that LRRK1 is indirectly associated with ULK1 via ATG13. Thus, LRRK1 immunoprecipitates with the entire endogenous ULK complex in response to mitophagy stimulation. Moreover, the interaction between LRRK1 and ATG13 induced by CCCP depended on PINK1 (Fig. S4D).

Next, we investigated whether the role of ATG13 for LRRK1 in Parkin-dependent mitophagy is only to recruit LRRK1 to the mitochondria. Thus, we examined whether ectopic recruitment of ATG13 to the mitochondria by CID could induce Rab7 Ser-72 phosphorylation. We found that the addition of rapalog to U2OS cells expressing FKBP–GFP–ATG13 and FRB–Fis1 failed to elicit Rab7 Ser-72 phosphorylation on the mitochondria decorated with GFP–ATG13 (Fig. 5C,D). Thus, the ectopic localization of ATG13 to the mitochondria cannot induce Rab7 phosphorylation. However, we found that the co-expression of the constitutively active LRRK1 mutant LRRK1(Y944F) in cells expressing FKBP–GFP–ATG13 and FRB–Fis1 could induce the localization of LRRK1(Y944F) to the GFP–ATG13-decorated mitochondria in a rapalog treatment-dependent manner, inducing the pS72-Rab7 signal on the mitochondria (Fig. 5E,F). By contrast, the co-expression of wild-type LRRK1 in cells expressing FKBP–GFP–ATG13 and FRB–Fis1 induced mitochondrial localization of wild-type LRRK1 upon the addition of rapalog, but not Rab7 Ser-72 phosphorylation on mitochondria (Fig. 5E,F). Furthermore, the interaction of overexpressed LRRK1(Y944F) with ATG13 was dependent on CCCP stimulation (Fig. S4E). These results suggest that ATG13 has two functions concerning LRRK1 in Parkin-mediated mitophagy – the recruitment of LRRK1 to damaged mitochondria and a role in mediating LRRK1 activation.

How does the ULK complex activate LRRK1 in Parkin-mediated mitophagy? ULK kinase might activate LRRK1 kinase activity through phosphorylation because LRRK1(Y944F) overexpression can suppress the ULK1 and ULK2 double-depletion phenotype (Fig. 3C,D). Therefore, we performed *in vitro* kinase assays using purified recombinant glutathione S-transferase (GST)-tagged ULK1. Immunopurified kinase-negative GFP–LRRK1(KM) was incubated with GST–ULK1 *in vitro*. The kinase-negative form of LRRK1 was used as a substrate to prevent autophosphorylation of LRRK1. We found that GST–ULK1 phosphorylated both itself and GFP–LRRK1(KM) (Fig. S6), indicating that this modification results from trans-phosphorylation. These results raise the possibility that mitophagy activation induces the recruitment of the ULK complex and LRRK1 to the damaged mitochondria, resulting in the activation of ULK kinase, thereby phosphorylating and activating LRRK1.

### Relationship between LRRK1 and ATG101 in Parkin-mediated mitophagy

**Fig. 6.**
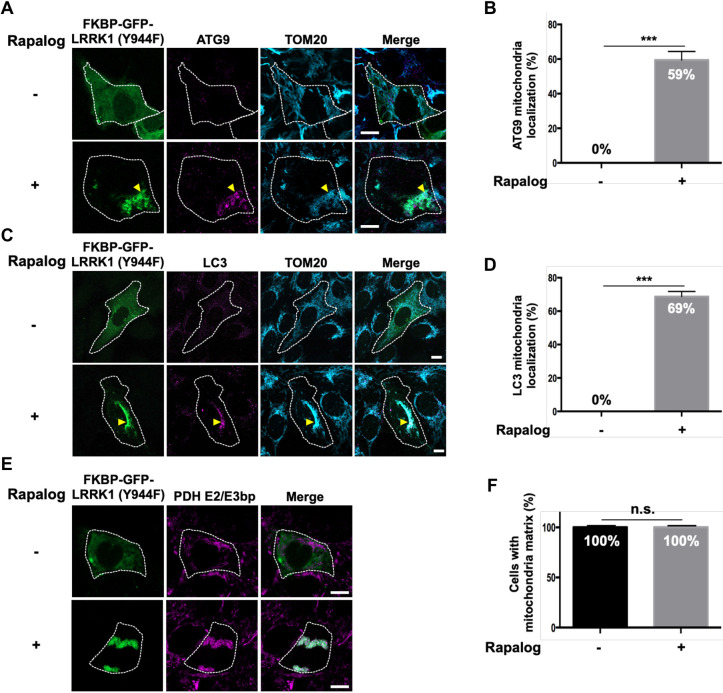
**Effects of artificially targeting LRRK1(Y944F) to mitochondria on mitophagy.** (A,C) Effect of artificially targeting LRRK1(Y944F) to mitochondria on the recruitment of ATG9 and LC3 to mitochondria. U2OS cells were co-transfected with FKBP–GFP–LRRK1(Y944F) and FRB–Fis1. Cells were treated with or without rapalog (0.5 µM) for 24 h and immunostained with antibodies against ATG9 (A), LC3 (C) and TOM20 (cyan), for mitochondria. White dotted lines indicate GFP–LRRK1(Y944F)-expressing cells. Yellow arrowheads indicate ATG9 or LC3 signals on mitochondria decorated with GFP–LRRK1(Y944F). Scale bars: 10 µm. (B,D) Quantification of the colocalization of TOM20 with ATG9 (B) or LC3 (D). Data represent the percentage of GFP–LRRK1(Y944F)-expressing cells with ATG9 or LC3 signals on mitochondria decorated with GFP–LRRK1(Y944F) per total GFP–LRRK1(Y944F)-expressing cells (*n*=3; >25 cells counted per condition). The error bars represent s.d. ***P<0.001 (Welch's *t*-test). (E) Effect of artificially targeting LRRK1(Y944F) to the mitochondria on the mitochondrial elimination. U2OS cells were co-transfected with FKBP–GFP–LRRK1(Y944F) and FRB–Fis1. Cells were treated with or without rapalog (0.5 µM) for 72 h and immunostained with the antibody against PDH E2/E3 bp (magenta), for the mitochondrial matrix. White dotted lines indicate GFP–LRRK1(Y944F)-expressing cells. Scale bars: 10 µm. (F) Quantification of mitochondrial clearance. Data represent the percentage of GFP–LRRK1(Y944F)-expressing cells with mitochondrial matrix per total GFP–LRRK1(Y944F)-expressing cells (*n*=3; >20 cells counted per condition). The error bars represent s.d. n.s., not significant (Welch's *t*-test).

Because ATG101 was not required for Rab7 Ser-72 phosphorylation on damaged mitochondria (Fig. 4A,B), we investigated whether ATG101 was needed for the clearance of damaged mitochondria. We found that ATG101 siRNA significantly reduced the percentage of cells lacking mitochondrial staining 24 h after O/A treatment (Fig. S7A–C). These results suggest that the ULK complex mediates mitophagy through LRRK1-dependent and LRRK1-independent pathways and that ATG101 engages in only the latter pathway. Consistent with this idea, the expression of LRRK1(Y944F) in ULK1 and ULK2 double-depleted cells suppressed the loss of Ser-72 phosphorylation in Rab7 on damaged mitochondria (Fig. 3C,D) but not the loss of depolarized mitochondrial clearance (Fig. S8A–C). Furthermore, when LRRK1(Y944F) was artificially targeted to the mitochondria by CID, ATG9 and LC3 were recruited to the mitochondria (Fig. 6A–D). In contrast, no clearance of mitochondrial matrix proteins was observed (Fig. 6E,F). Thus, the localization of activated LRRK1 to the mitochondria can trigger up to the step of mitochondrial recruitment of ATG9 and LC3.

### Relationship between LRRK1 and TBK1 in CCCP-induced Rab7 Ser-72 phosphorylation

**Fig. 7.**
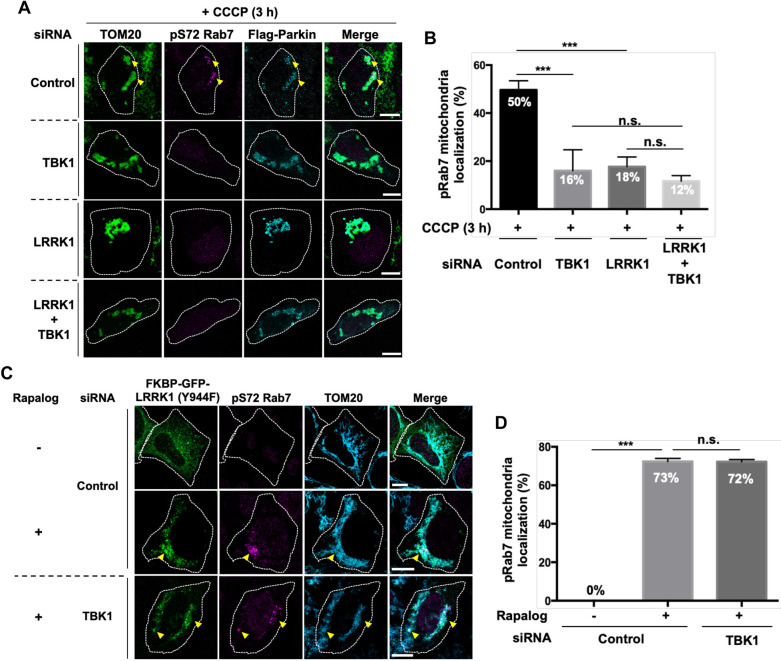
**Relationship between TBK1 and LRRK1 in CCCP-induced Rab7 Ser-72 phosphorylation.** (A) Effect of TBK1 depletion on the phosphorylation of Rab7 Ser-72. U2OS cells treated with control siRNA, TBK1 siRNA or LRRK1 siRNA were transfected with Flag–Parkin, as indicated. After 3 h of CCCP treatment (10 µM), the cells were immunostained with antibodies against TOM20 (green) for mitochondria, pS72-Rab7 (magenta), and Flag (cyan) for Parkin. White dotted lines indicate Flag–Parkin-expressing cells. Yellow arrowheads arrows indicate pS72-Rab7 signals on mitochondria decorated with Flag–Parkin. Scale bars: 10 µm. (B) Quantification of the colocalization of pSer-72 Rab7 and Flag–Parkin. Data represent the percentage of Flag–Parkin-expressing cells with pS72 Rab7 signals on structures decorated with Flag–Parkin per total Flag–Parkin-expressing cells (*n*=3; >40 cells counted per condition). The error bars represent s.d. ***P<0.001, n.s., not significant (Dunnett's multiple-comparison test). (C) Effect of artificially targeting LRRK1(Y944F) to the mitochondria on Rab7 Ser-72 phosphorylation deficiency due to TBK1 depletion. U2OS cells treated with control siRNA or TBK1 siRNA were co-transfected with FKBP–GFP–LRRK1(Y944F) and FRB–Fis1. Cells were treated with or without rapalog (0.5 µM) for 24 h and immunostained with antibodies against pS72-Rab7 (magenta) and TOM20 (cyan), for mitochondria. White dotted lines indicate GFP–LRRK1(Y944F)-expressing cells. Yellow arrowheads indicate the pS72-Rab7 signal on mitochondria decorated with GFP–LRRK1(Y944F). Scale bars: 10 µm. (D) Quantification of the colocalization of pSer-72 Rab7 and TOM20. Data represent the percentage of GFP–LRRK1(Y944F)-expressing cells with pS72 Rab7 signals on mitochondria decorated with GFP–LRRK1(Y944F) per total GFP–LRRK1(Y944F)-expressing cells (*n*=3; >20 cells counted per condition). The error bars represent s.d. ***P<0.001, n.s., not significant (Dunnett's multiple-comparison test).

Recent studies have demonstrated that TBK1 activation occurs early in Parkin-mediated mitophagy and that TBK1 directly phosphorylates Rab7 on Ser-72 *in vitro* (Heo et al., 2018). However, our results showed that LRRK1 phosphorylates Rab7 Ser-72 during Parkin-mediated mitophagy. Therefore, we evaluated the functional relationship between TBK1 and LRRK1 in the phosphorylation of Rab7 Ser-72 seen upon CCCP treatment. The depletion of TBK1 in Flag–Parkin-expressing cells significantly reduced the mitochondrial pS72-Rab7 signal induced by CCCP (Fig. 7A,B; Fig. S1E). This defect was comparable to that caused by LRRK1 knockdown (Fig. 7A,B). Furthermore, we found that the depletion of LRRK1 and TBK1 did not enhance the reduction of the mitochondrial pS72-Rab7 signal (Fig. 7A,B), suggesting that TBK1 and LRRK1 function in the same pathway. If LRRK1 functions downstream of TBK1, then the constitutively active LRRK1(Y944F) mutation would compensate for the loss of Rab7 phosphorylation caused by TBK1 knockdown. We found that the tethering of LRRK1(Y944F) to the mitochondria by CID triggered Rab7 Ser-72 phosphorylation in TBK1-knockdown cells (Fig. 7C,D). These results indicate that TBK1 does not directly phosphorylate Rab7 Ser-72 *in vivo* in Parkin-mediated mitophagy.

## DISCUSSION

Given that LRRK1 is a regulator of autophagy (Toyofuku et al., 2015), we aimed to investigate the role of LRRK1 in Parkin-dependent mitophagy. Here, we identified a novel signaling pathway, in which the activation of the ULK complex by mitochondrial depolarization leads to the phosphorylation and activation of LRRK1, followed by Rab7 phosphorylation and elimination of impaired mitochondria. This study revealed that LRRK1 is important for Parkin-mediated mitophagy.

Our results highlight the functional significance of the ULK complex in the regulation of LRRK1 in Parkin-mediated mitophagy. The ULK complex consists of ULK1 or ULK2, FIP200, ATG13 and ATG101 (Hurley and Young, 2017). We showed that ectopic placement of active LRRK1(Y944F) on the mitochondria induces Rab7 Ser-72 phosphorylation in ATG13-depleted cells. This indicates that direct targeting of LRRK1(Y944F) to the mitochondria can bypass the ATG13 requirement for Ser-72 phosphorylation in Rab7. Thus, mitochondrial localization of LRRK1 is essential for its function in this pathway. We found that LRRK1 forms a complex with ATG13 in a manner dependent on mitophagy activation, suggesting that ATG13 targets LRRK1 to damaged mitochondria. ATG13 binds to ULK1 and/or ULK2 and FIP200, potentially serving as a bridge between ULK1/2 and FIP200 within the complex (Hurley and Young, 2017). Therefore, subunits of this complex might serve as scaffolding for the recruitment of LRRK1. Indeed, it is known that FIP200 mediates the recruitment of the ULK complex to the mitochondrial surface (Vargas et al., 2019).

How is LRRK1 activated in response to mitochondrial depolarization? In this study, we showed that the expression of the activated mutant LRRK1(Y944F) rescues the defect in CCCP-induced phosphorylation of Rab7 at Ser-72 caused by ULK1 and ULK2 double-depletion. Furthermore, we demonstrated that ULK1 phosphorylates LRRK1, suggesting that ULK is involved in LRRK1 activation through phosphorylation. In contrast, the ectopic recruitment of ATG13 alone to the mitochondria cannot induce Rab7 Ser-72 phosphorylation. However, the co-expression of LRRK1(Y944F), but not wild-type LRRK1, in cells expressing mitochondria-targeted ATG13 can induce the pS72-Rab7 signal on mitochondria. These results provide insights into the mechanism through which the ULK complex plays a role in LRRK1 activation in Parkin-mediated mitophagy. Therefore, mitophagy activation induces the recruitment of the ULK complex and LRRK1 to the damaged mitochondria, where ULK kinase is activated by local clustering, thereby phosphorylating and activating LRRK1. Thus, the localization of LRRK1 on the mitochondria by the ULK complex indicates the LRRK1 activation is linked to spatial regulation. This model simultaneously resolves the timing and location of Parkin-mediated mitophagy induction.

**Fig. 8.**
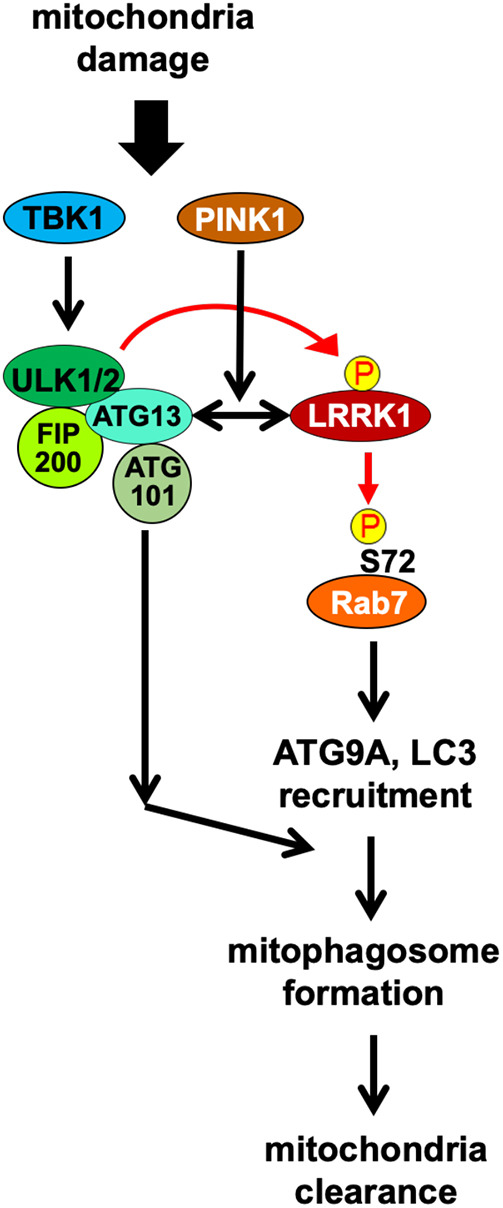
**Schematic model for the regulation of Parkin-mediated mitophagy by the ULK complex–LRRK1 axis.** The ULK complex mediates mitophagy through LRRK1-dependent and LRRK1-independent pathways. However, ATG101 is involved only in the latter pathway. The LRRK1-independent pathway feeds into the downstream events of the mitophagosome biogenesis mediated by the LRRK1-dependent pathway.

In this study, we found that in Parkin-mediated mitophagy, ATG101 is required for the clearance of depolarized mitochondria but not for Rab7 Ser-72 phosphorylation on depolarized mitochondria. These results suggest that the ULK complex mediates mitophagy through both LRRK1-dependent and LRRK1-independent pathways, whereas ATG101 is involved in only the latter pathway (Fig. 8). Accordingly, the expression of the active mutant LRRK1(Y944F) in ULK1 and ULK2 double-depleted cells can suppress the loss of Rab7 Ser-72 phosphorylation on damaged mitochondria but not the loss of clearance of depolarized mitochondria. When LRRK1(Y944F) is ectopically localized to the mitochondria, ATG9 and LC3 are translocated to the mitochondria decorated with LRRK1(Y944F), but the mitochondrial matrix is not removed. Thus, the activation of the LRRK1 pathway on the mitochondrial surface induces Rab7 Ser-72 phosphorylation and mitochondrial recruitment of ATG9 and LC3. This finding is consistent with the fact that Ser-72 phosphorylation in Rab7 is required for ATG9 recruitment (Heo et al., 2018). These results suggest that in Parkin-mediated mitophagy, the LRRK1-independent pathway mediated by the ULK complex feeds into the downstream events of mitophagosome biogenesis mediated by the LRRK1-dependent pathway (Fig. 8).

Recently, Heo et al. (2018) reported that TBK1 promoted mitophagy by phosphorylating Rab7 on Ser-72. Thus, TBK1 was proposed to be the kinase responsible for Rab7 Ser-72 phosphorylation in Parkin-mediated mitophagy. However, in TBK1-depleted cells, mitochondrial targeting of active LRRK1 kinase induces Rab7 phosphorylation on the mitochondria. This indicates that mitochondrial localization of LRRK1 bypasses the function of TBK1. In addition, the effect of TBK1 siRNA on the loss of mitochondrial Rab7 Ser-72 phosphorylation is not enhanced by LRRK1 siRNA. These results suggest that TBK1 and LRRK1 function in the same pathway to regulate Rab7 phosphorylation. Based on these findings, we propose that LRRK1 functions downstream of TBK1 to regulate mitophagy. Furthermore, recent studies have shown that TBK1 phosphorylates the autophagy receptor NDP52 and facilitates the binding of NDP52 to FIP200, resulting in the recruitment of the ULK complex to the mitochondrial surface (Vargas et al., 2019). Given that artificial localization of ULK1 to the mitochondria triggers mitophagy even in the absence of TBK1 (Vargas et al., 2019), TBK1 functions primarily upstream of the ULK complex in Parkin-dependent mitophagy. Therefore, it is likely that TBK1 does not directly phosphorylate Rab7 at Ser-72 during mitophagy.

What is the role of Rab7 Ser-72 phosphorylation in Parkin-mediated mitophagy? When Rab7 is recruited to damaged mitochondria in response to mitochondrial depolarization, it plays a critical role in correctly targeting ATG9-bearing vesicles to induce mitophagosome formation around damaged mitochondria (Jimenez-Orgaz et al., 2018; Yamano et al., 2018). Moreover, cells expressing non-phosphorylatable Rab7(S72A) are defective in the accumulation of ATG9 on mitochondria, suggesting that Rab7 Ser-72 phosphorylation plays a significant role in the recruitment of ATG9 (Heo et al., 2018). The Ser-72 residue is located in the switch-II region of the Rab7 GTPase domain, and this region participates in interactions with effectors or regulatory proteins when Rab7 is in its GTP-bound form (Pfeffer, 2005). Phosphorylation of the corresponding residues in Rab GTPases alters their binding to partner proteins (Steger et al., 2016, 2017). Indeed, we recently demonstrated that Rab7 phosphorylation at Ser-72 by LRRK1 increases the association of Rab7 with its effector RILP, resulting in enhanced transport of EGFR-containing endosomes (Hanafusa et al., 2019). Our findings reveal a mechanism by which LRRK1 determines the selective interaction between Rab7 and its effectors in a cargo-dependent manner. These results lead to the hypothesis that Rab7 Ser-72 phosphorylation regulates its interaction with the effector involved in ATG9 recruitment.

LRRK2 engages in the regulation of mitochondrial homeostasis in a manner dependent on its kinase and pathogenic mutations (Bonello et al., 2019). LRRK2 functions as a negative regulator of mitophagy by phosphorylating Rab10 at Thr-73, a residue corresponding to Rab7 Ser-72 (Wauters et al., 2019). Rab10 accumulates on depolarized mitochondrial surfaces in a Parkin-dependent manner and promotes mitophagy by recruiting the autophagy receptor OPTN. Phosphorylation of Rab10 at Thr-73 by pathogenic LRRK2 mutants reduces binding to OPTN and results in impaired mitophagy. Thus, LRRK1 and LRRK2 have opposite effects on Parkin-mediated mitophagy; LRRK1 and LRRK2 act as positive and negative regulators by phosphorylating Rab7 and Rab10, respectively. This might explain why LRRK2 mutations are detected in patients with familial PD, whereas LRRK1 mutations are not.

## MATERIALS AND METHODS

### Cell culture, antibodies and reagents

U2OS and HEK293 cells were cultured in DMEM containing 10% fetal bovine serum (042-30555, FUJIFILM Wako). These cell lines were obtained from either the American Type Culture Collection or the Japanese Collection of Research Bioresources and were regularly evaluated for *Mycoplasma* contamination. The antibodies and their suppliers were as follows: anti-TOM20 (F-10, Santa Cruz Biotechnology), anti-GFP (598, MBL), anti-Flag (M2, Sigma-Aldrich or FLA-1, MBL), anti-Myc (9E10, Santa Cruz Biotechnology), anti-HA (16B12, Babco), anti-ATG9 (Abcam), anti-LC3 (M152-3, MBL), anti-C-III core 1 (Invitrogen), anti-ATG13 (M183-3, MBL), anti-ULK1 (D8H5, Cell Signaling Technology or A7481, Sigma-Aldrich), anti-TBK1 (E8I3G, CST), anti-ATG101 (Abcam) and anti-PDH E2/E3bp (Abcam). Affinity-purified rabbit antibodies against pS72-Rab7 were produced according to a previously described method (Hanafusa et al., 2019). CCCP and antimycin A were purchased from Sigma-Aldrich. Oligomycin was purchased from Merck Millipore.

### Plasmids, mutations, and RNA interference

GFP–LRRK1, GFP–LRRK1(K1243M), GFP–LRRK1(Y944F) and pHAGE-mt-mKeima-P2A-FRB-Fis1 were generated as previously described (Hanafusa et al., 2011; Vargas et al., 2019). GFP–LRRK1(Y944F) and GFP–ATG13 were subcloned into the pHAGE-FKBP vector (Dr Richard Youle, NINDS, Bethesda, MD). pEGFP-hATG13 was Addgene plasmid #22875 (deposited by Noboru Mizushima). siRNA-resistant LRRK1 was generated as previously described (Hanafusa et al., 2011). siRNA for human LRRK1 [target sequence: 5′-GCAGGAACAGGAAAGTCACCATTTA(TT)-3′], human ULK1 [target sequence: 5′-CGCCTGTTCTACGAGAAGA(TT)-3′], human ULK2 [target sequence: 5′-GCTCGTTACCTACATAGTA(TT)-3′], human TBK1 [target sequence: 5′-GGTTTGGCTCTTTAACCAT(TT)-3′], human ATG13 [target sequence: 5′-GCATTCATGTCTACCAGGCAATTTG(TT)-3′], human PINK1 [target sequence: 5′-AAGCCATCTTGAACACAAT(TT)-3′], and human ATG101 [target sequence: 5′-GACTGTGACTTCATCGACTTCACTT(TT)-3′] were purchased from JBioS. Control siRNA (Silencer Select; Life Technologies) was used as a negative control. Annealed siRNAs were transfected using RNAiMAX (Invitrogen). Then, transfected cells were analyzed 72 h after transfection.

### *In vitro* kinase assay

The recombinant protein GST–ULK1 was purchased from SignalChem. GFP–LRRK1 proteins were expressed in HEK293 cells and immunopurified using an anti-GFP antibody (Ishikawa et al., 2012). Kinase reactions were performed in a final volume of 20 µl containing the following reagents: 50 mM HEPES pH 7.4, 5 mM MgCl_2_, 0.5 mM DTT, 5 µCi [γ-^32^P]ATP and 100 µM ATP. Samples were incubated for 20 min at 30°C, and the reactions were terminated by the addition of Laemmli sample buffer, followed by boiling. Samples were resolved by SDS-PAGE and analyzed using autoradiography.

### Immunoprecipitation

Cells were lysed in RIPA buffer [50 mM Tris-HCl, pH 7.4, 0.15 M NaCl, 0.25% deoxycholic acid, 1% NP-40, 1 mM EDTA, 1 mM dithiothreitol, phosphatase inhibitor cocktail 2 (Sigma-Aldrich), and protease inhibitor cocktail (Sigma-Aldrich)], followed by centrifugation at 15,000* **g*** for 12 min. The supernatant was added to 50 µl (1.5 mg) of Dynabeads Protein G (Invitrogen) with the indicated antibodies (10 µg) and rotated for 2 h at 4°C. The beads were then washed three times with ice-cold phosphate-buffered saline and subjected to immunoblotting. Raw data for western blots are provided in Fig. S9.

### Immunofluorescence and image analysis

For immunofluorescence staining, the cells were grown on coverslips, treated as indicated, fixed in 4% paraformaldehyde for 15 min at 37°C or methanol for 2 min at −20°C, and permeabilized in 0.5% Triton X-100 for 5 min. Subsequently, the cells were incubated with primary and secondary antibodies. The primary antibodies used were as follows: mouse anti-Flag at 1:500, anti-Myc at 1:200, rabbit anti-TOM20 at 1:200, anti-pS72-Rab7 at 1:250, anti-ATG9 at 1:100, anti-LC3 at 1:500, anti-C-III core 1 at 1:100, and anti-PDH E2/E3bp at 1:500. The secondary antibodies used were as follows: Alexa-Fluor 488-, 555-, or 647-goat anti-mouse IgG or anti-rabbit IgG antibodies (Invitrogen). Confocal microscopy was performed using a Zeiss LSM800 microscope. In quantifying the percentage of cells with pS72-Rab7 or PDH E2/E3bp signals in Flag–Parkin-decorated structures, GFP–LRRK1(Y944F)-decorated structures, GFP–ATG13-decorated structures or TOM20-positive mitochondria, the colocalization of these signals in immunofluorescence images was manually counted. For each series of experiments, the microscopic settings were optimized for the brightest unsaturated images and maintained during the analysis.

### Quantification of mitochondrial mass

Mitochondrial clearance was quantified using ImageJ (National Institutes of Health, Bethesda, MD, USA). First, the region of the cell to be analyzed was outlined with a closed polygon, and the immunofluorescence intensity of C-III core 1 within this region was determined. Then, the net fluorescence intensity of each was calculated by subtracting the background immunofluorescence. The relative C-III core 1 fluorescence intensity for each cell was obtained by dividing the value of C-III core 1 immunofluorescence by the region. Cells with a signal of 0 were defined as cells with mitochondrial clearance and summarized in a bar graph.

### Statistical analysis

Statistical analysis was conducted using either Dunnett's multiple-comparison test or Welch's *t*-test. Data are combined from three independent experiments. Error bars represent standard deviation (s.d.). Detailed *n* values for each figure panel are stated in the corresponding legends. No statistical method was employed to predetermine the sample size. GraphPad Prism was used for the analysis of statistical significance.

## Supplementary Material

10.1242/joces.260395_sup1 Supplementary information
