# Supplementary material for: The ULK complex–LRRK1 axis regulates Parkin-mediated mitophagy via Rab7 Ser-72 phosphorylation
Source: J Cell Sci. 2022 Dec 7;135(23):jcs260395. doi: 10.1242/jcs.260395 (PMC9789397; doi:10.1242/jcs.260395)
Supplement: Supplementary information [file joces-135-260395-s1.pdf]

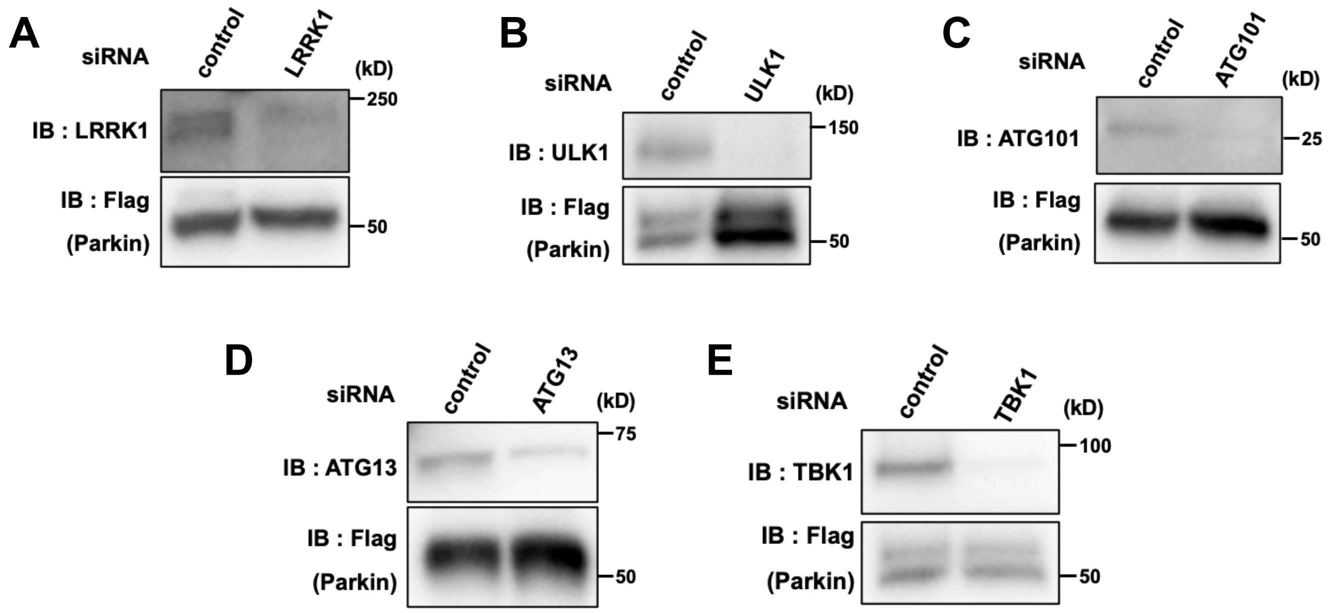

Fig. S1. Effect of siRNA.
(A–E) U2OS cells expressing Flag-Parkin were treated with control siRNA, LRRK1 siRNA **(A)**, ULK1 siRNA **(B)**, ATG101 siRNA **(C)**, ATG13 siRNA **(D)**, or TBK1 siRNA **(E)**. Total lysates were immunoblotted (IB) with antibodies as indicated. Flag-Parkin is served as the loading control.

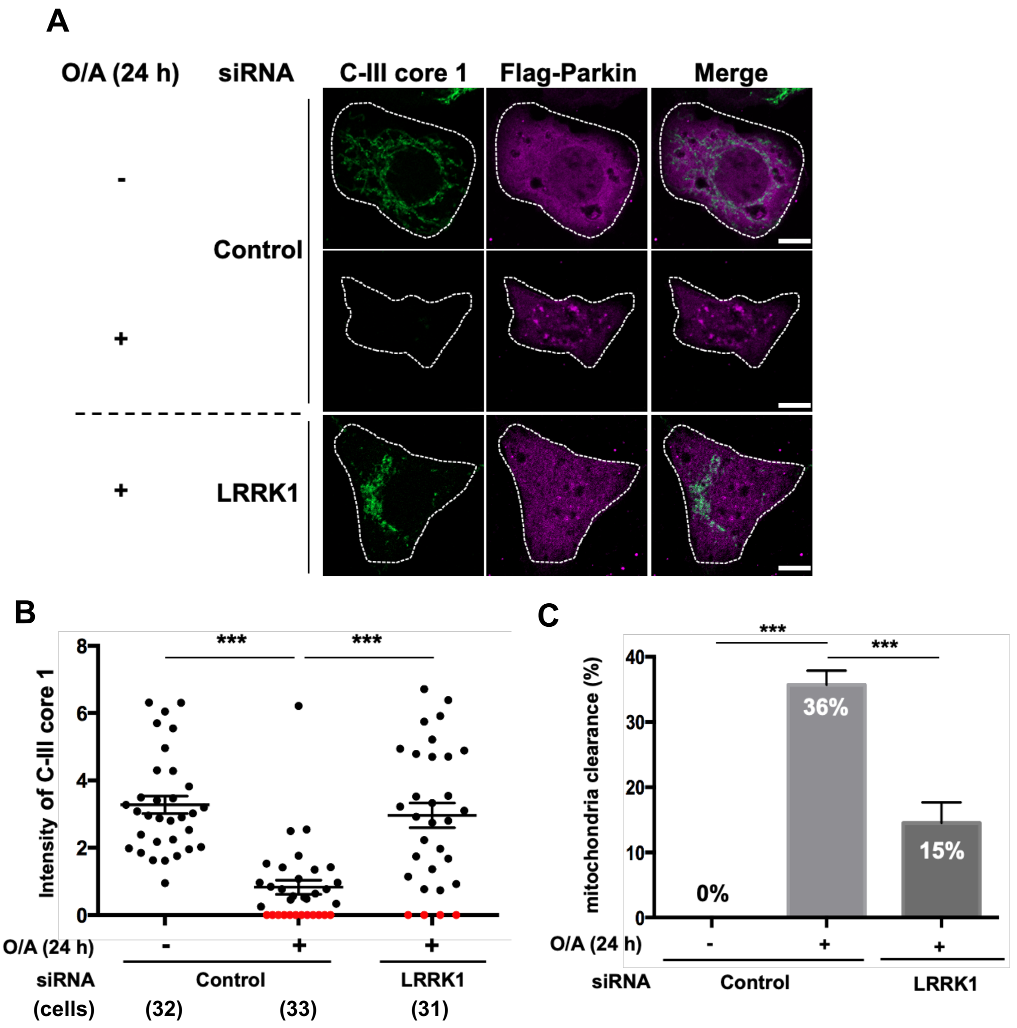

Fig. S2. Effect of LRRK1 depletion on the elimination of depolarized mitochondria.

(A) U2OS cells treated with either control siRNA or LRRK1 siRNA were transfected with Flag-Parkin. After 24 h of treatment with or without O/A (a combination of oligomycin 10 µM and antimycin A 10 µM), the cells were immunostained with the following antibodies: C-III core 1 (magenta) for the IMM and mitochondrial matrix and Flag (cyan) for Parkin. White dotted lines indicate Flag-Parkin-expressing cells. Scale bar: 10 µm.

(B) Quantification of mitochondrial mass. Data were plotted as the fluorescence intensity of C-III core 1 in Flag-Parkin-expressing cells after background subtraction. The number of cells examined is indicated. Red circles indicate cells with mitochondria clearance. A typical example of an experiment conducted three times is shown. The error bars represent SD. ***$P < 0.001$, n.s., not significant (Dunnett's multiple-comparison test).

(C) Quantification of mitochondrial clearance. Data represent the percentage of Flag-Parkin-expressing cells without mitochondrial matrix per total Flag-Parkin-expressing cells (n = 3; >30 cells counted per condition). The error bars represent SD. ***P < 0.001 (Dunnett's multiple-comparison test).

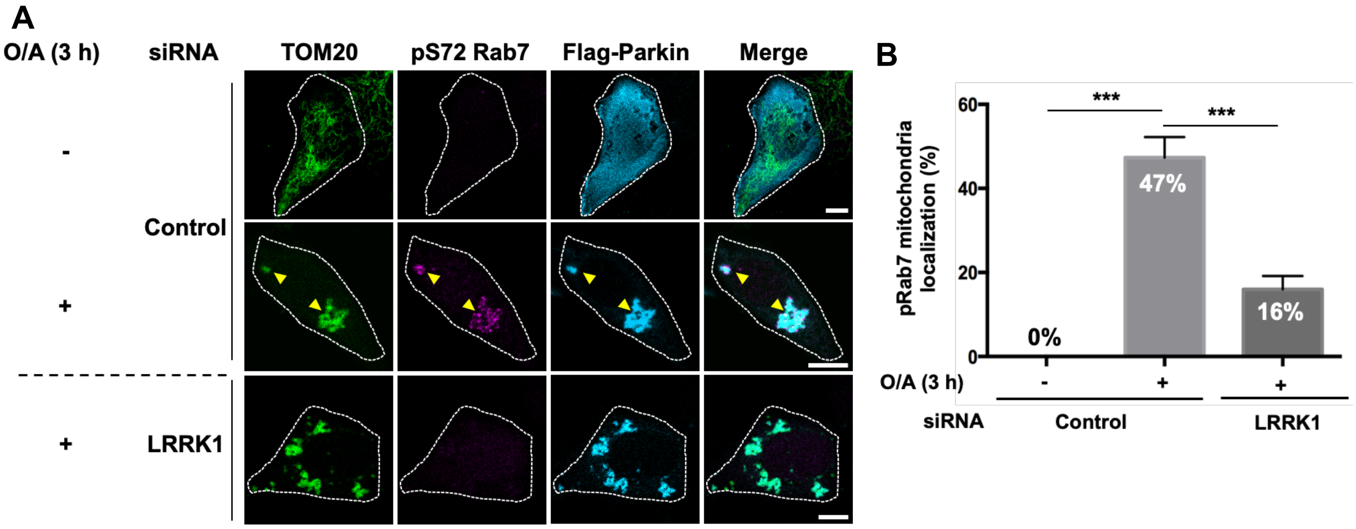

Fig. S3. Effect of LRRK1 depletion on mitochondrial Rab7 Ser-72 phosphorylation.
(A) U2OS cells treated with either control siRNA or LRRK1 siRNA were transfected with Flag-Parkin. After 3 h of treatment with or without O/A (a combination of oligomycin 10 μM and antimycin A 10 μM), the cells were immunostained with the following antibodies: TOM20 (green) for mitochondria, pS72-Rab7 (magenta), and Flag (cyan) for Parkin. White dotted lines indicate Flag-Parkin-expressing cells. Yellow arrowheads indicate the pS72-Rab7 signal on mitochondria decorated with Flag-Parkin. Scale bar: 10 μm.
(B) Quantification of the co-localization of pSer-72 Rab7 and Flag-Parkin. Data represent the percentage of Flag-Parkin-expressing cells with pS72 Rab7 signals on structures decorated with Flag-Parkin per total Flag-Parkin-expressing cells (n = 3; >30 cells counted per condition). The error bars represent SD. ***$P < 0.001$ (Dunnett's multiple-comparison test).

Journal of Cell Science • Supplementary information

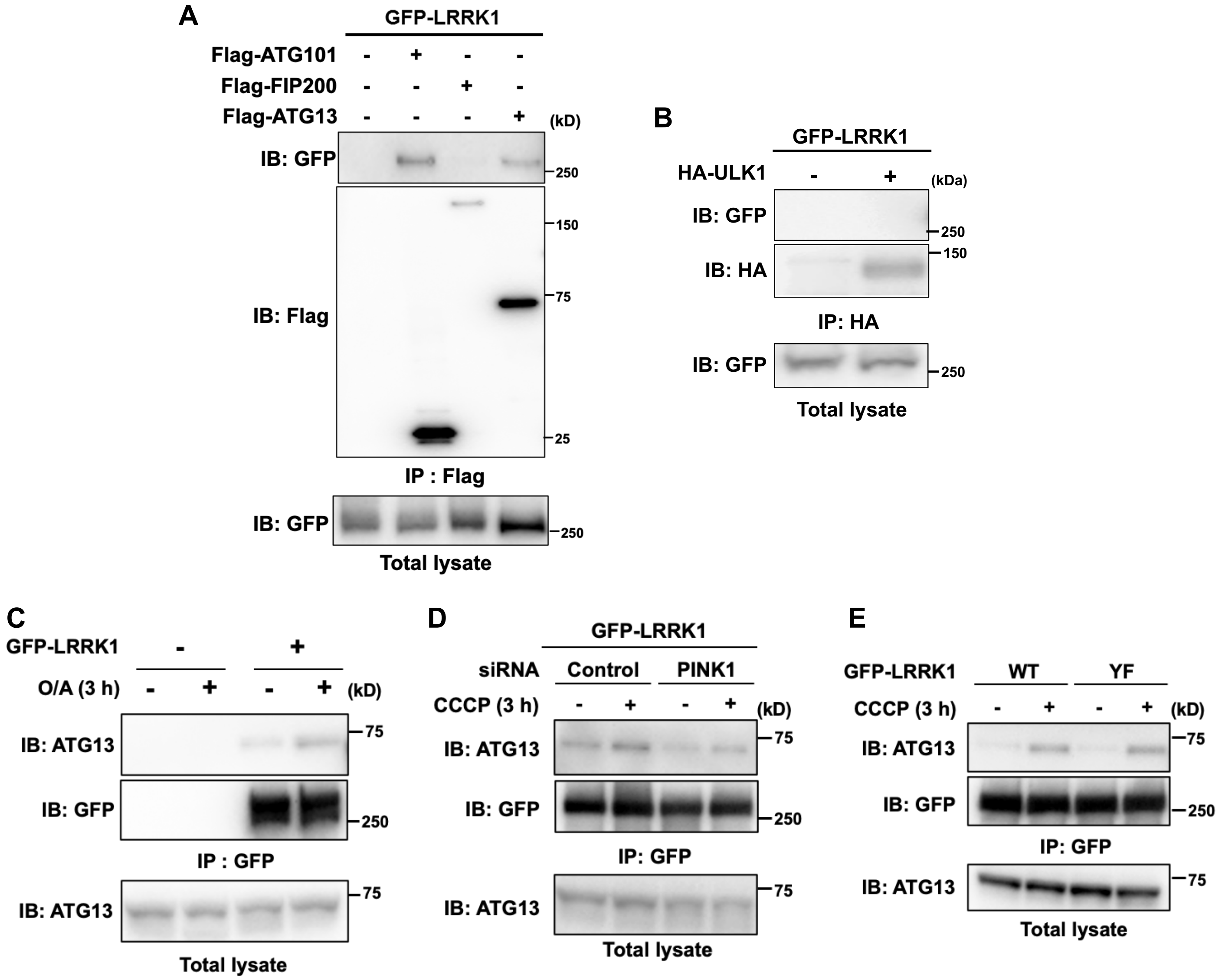

Fig. S4. Interactions of LRRK1 with ATG13, ATG101, FIP200, or ULK1.

(A) HEK293 cells were co-transfected with GFP-LRRK1 and Flag-ATG101, Flag-FIP200, or Flag-ATG13, as indicated. A complex formation was detected by immunoprecipitation (IP) with anti-Flag antibodies, followed by immunoblotting (IB) with antibodies as indicated.

(B) HEK293 cells were co-transfected with GFP-LRRK1 and HA-ULK1, as indicated. A complex formation was detected by immunoprecipitation (IP) with anti-HA antibodies, followed by immunoblotting (IB) with anti-GFP antibodies as indicated.

(C) HEK293 cells were transfected with GFP-LRRK1. Cells were treated with or without O/A (a combination of oligomycin 10 µM and antimycin A 10 µM) for 3 h. Complex formation was detected by immunoprecipitation (IP) with anti-GFP antibodies, followed by immunoblotting (IB) with antibodies as indicated.

(D) Effect of PINK1 depletion on the LRRK1–ATG13 interaction. HEK293 cells treated with control siRNA or PINK1 siRNA were transfected with GFP-LRRK1. Cells were treated with or without CCCP for 3 h. Complex formation was detected by immunoprecipitation (IP) with anti-GFP antibodies, followed by immunoblotting (IB) with antibodies, as indicated.

(E) Effect of LRRK1 activity on the LRRK1–ATG13 interaction. HEK293 cells were transfected with GFP-LRRK1 (wild-type or Y944F). Cells were treated with or without CCCP for 3 h. Complex formation was detected by immunoprecipitation (IP) with anti-GFP antibodies, followed by immunoblotting (IB) with antibodies, as indicated.

Journal of Cell Science • Supplementary information

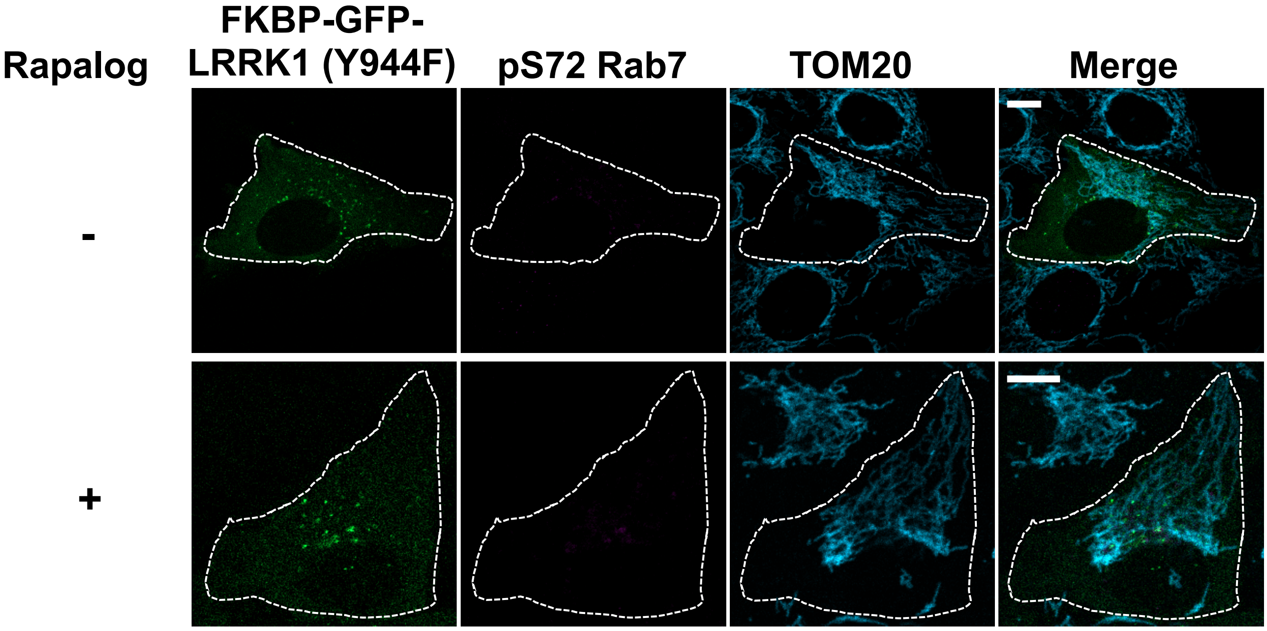

Fig. S5. Requirement of FRB-Fis1 for the recruitment of FKBP-GFP-LRRK1(Y944F) to mitochondria.

U2OS cells were transfected with FKBP-GFP-LRRK1(Y944F). The cells were treated with or without rapalog (0.5 µM) for 24 h and immunostained with the following antibodies: pS72-Rab7 (magenta) and TOM20 (cyan) for mitochondria. White dotted lines indicate GFP-LRRK1(Y944F)-expressing cells. Scale bar: 10 µm.

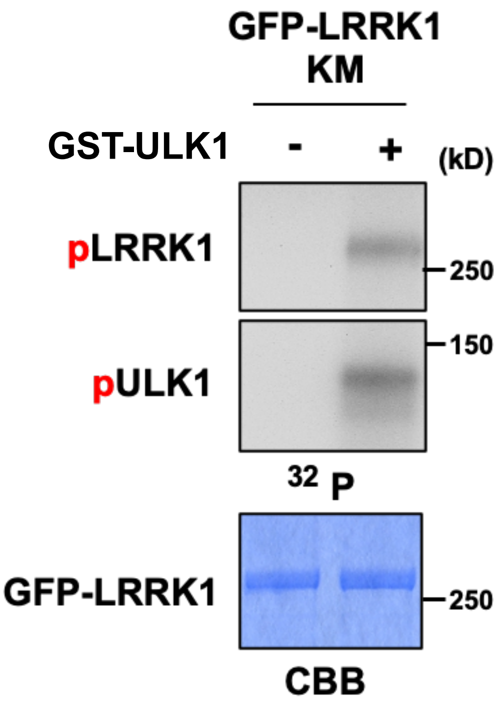

Fig. S6. ULK1 phosphorylates LRRK1 in vitro.
HEK293 cells were transfected with GFP-LRRK1(KM), and cell lysates were immunoprecipitated with anti-GFP antibodies. Immune-purified LRRK1 protein was incubated with recombinant GST-ULK1 in the presence of [γ-^{32}P] ATP for 20 min at 30° C. Autophosphorylated ULK1 and phosphorylated LRRK1 were resolved by SDS-PAGE (^{32}P). Protein input was confirmed using Coomassie Brilliant Blue (CBB) staining.

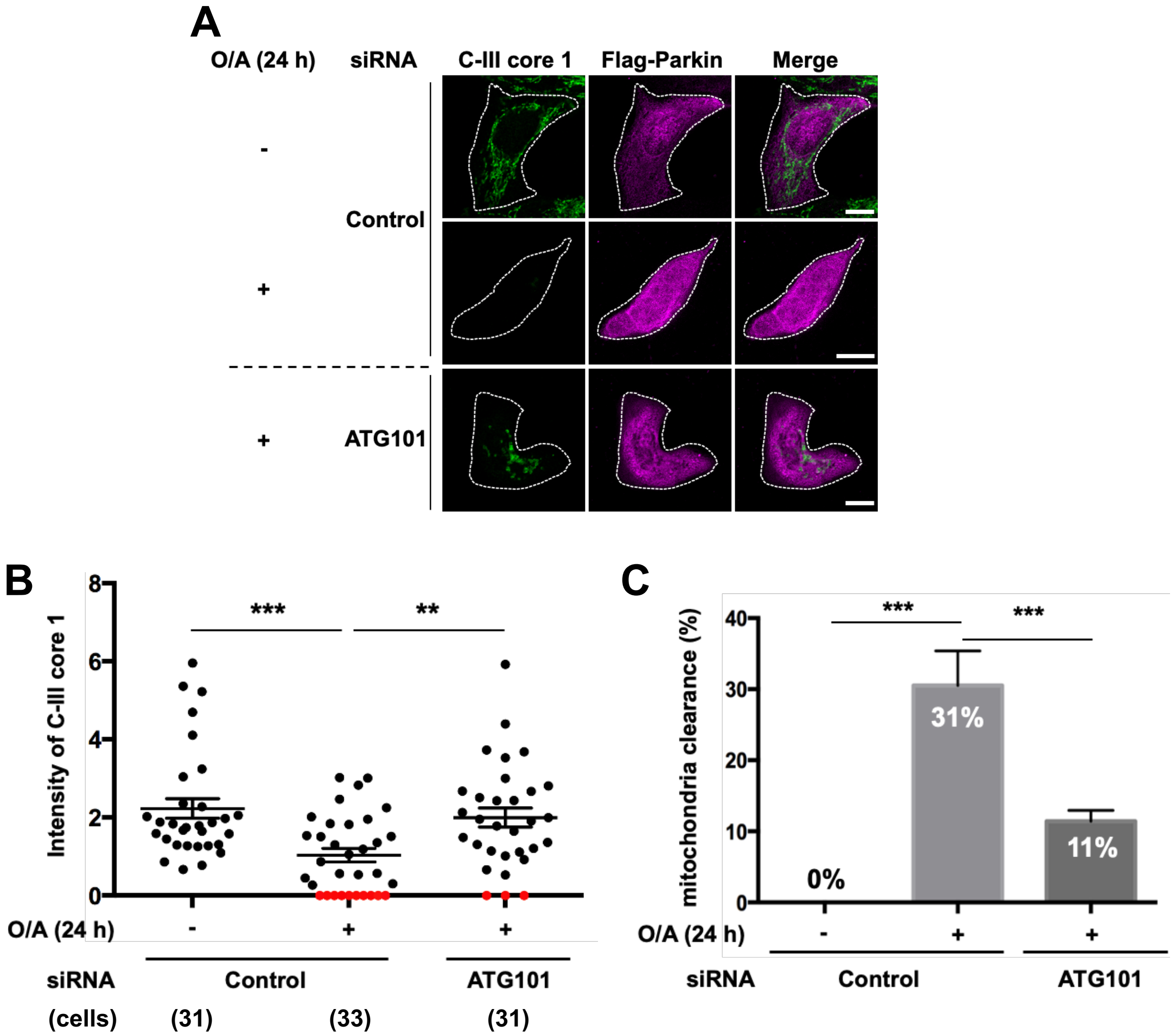

Fig. S7. Effect of ATG101 depletion on the elimination of depolarized mitochondria.

(A) U2OS cells treated with either control siRNA or ATG101 siRNA were transfected with Flag-Parkin. After 24 h of treatment with or without O/A (a combination of oligomycin 10 μM and antimycin A 10 μM), the cells were immunostained with the following antibodies: C-III core 1 (magenta) for the IMM and mitochondrial matrix and Flag (cyan) for Parkin. White dotted lines indicate Flag-Parkin-expressing cells. Scale bar: 10 μm.

(B) Quantification of mitochondrial mass. Data were plotted as the fluorescence intensity of C-III core 1 in Flag-Parkin-expressing cells after background subtraction. The number of cells examined is indicated. Red circles indicate cells with mitochondria clearance. A typical example of an experiment conducted three times is shown. The error bars represent SD. $^{**}P < 0.01$, $^{***}P < 0.001$ (Dunnett's multiple-comparison test).

(C) Quantification of mitochondrial clearance. Data represent the percentage of Flag-Parkin-expressing cells without mitochondrial matrix per total Flag-Parkin-expressing cells (n = 3; >30 cells counted per condition). The error bars represent SD. $^{***}P < 0.001$ (Dunnett's multiple-comparison test).

Journal of Cell Science • Supplementary information

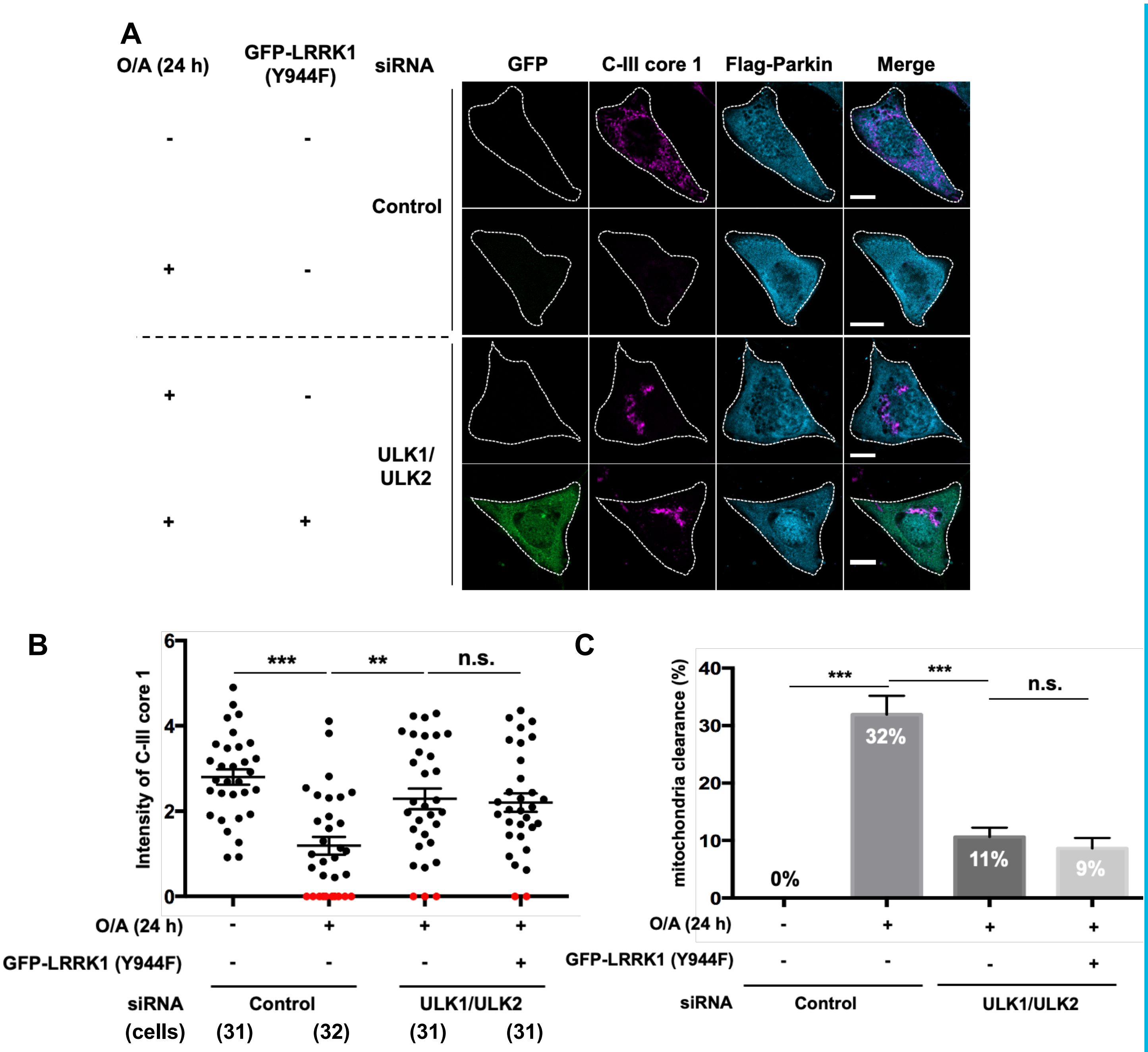

Fig. S8. Relationship between LRRK1 and ULK1/ULK2 in the elimination of depolarized mitochondria.

(A) Effect of LRRK1(Y944F) on the mitochondrial clearance defect due to ULK1/ULK2 depletion. U2OS cells treated with control siRNA or ULK1/ULK2 siRNAs were transfected with Flag-Parkin and GFP-LRRK1(Y944F), as indicated. After 24 h of treatment with or without O/A (a combination of oligomycin 10 µM and antimycin A 10 µM), the cells were immunostained with the following antibodies: C-III core 1 (magenta) for the IMM and mitochondrial matrix and Flag (cyan) for Parkin. White dotted lines indicate Flag-Parkin-expressing cells. Scale bar: 10 µm.

(B) Quantification of mitochondrial mass. Data were plotted as the fluorescence intensity of C-III core 1 in Flag-Parkin-expressing cells after background subtraction. The number of cells examined is indicated. Red circles indicate cells with mitochondria clearance. A typical example of an experiment conducted three times is shown. The error bars represent SD. $^{**}P < 0.01$, $^{***}P < 0.001$, n.s., not significant (Dunnett's multiple-comparison test).

(C) Quantification of mitochondrial clearance. Data represent the percentage of Flag-Parkin-expressing cells without mitochondrial matrix per total Flag-Parkin-expressing cells (n = 3; >30 cells counted per condition). The error bars represent SD. $^{***}P < 0.001$, n.s., not significant (Dunnett's multiple-comparison test).

Journal of Cell Science • Supplementary information

Fig. 2E

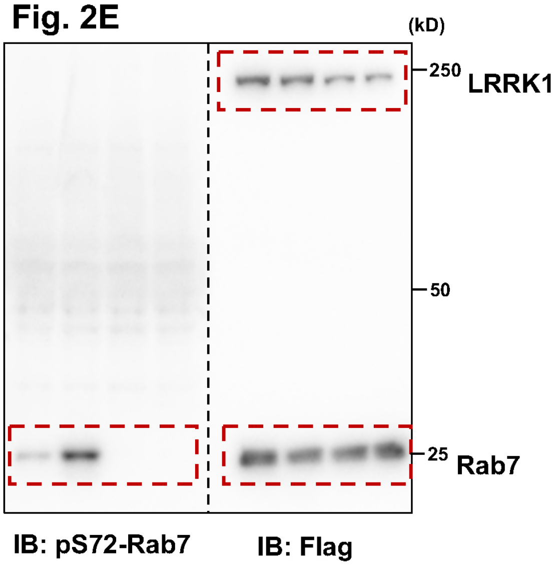

Fig. 5A

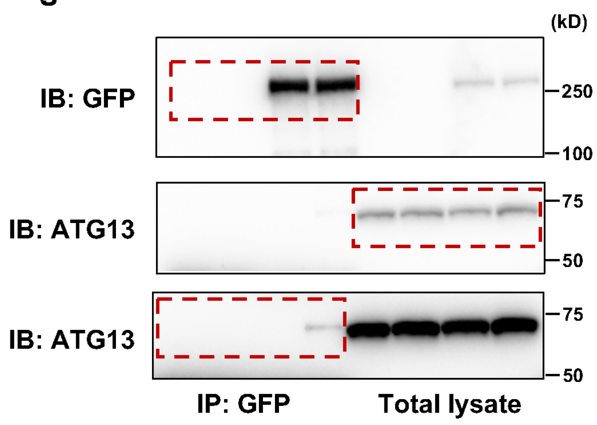

Fig. 5B

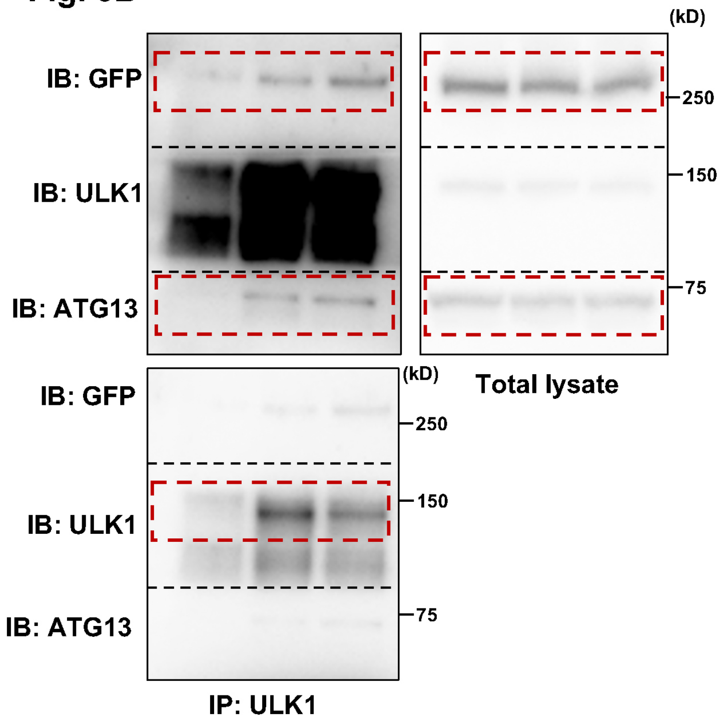

Journal of Cell Science • Supplementary information

Fig. S4A

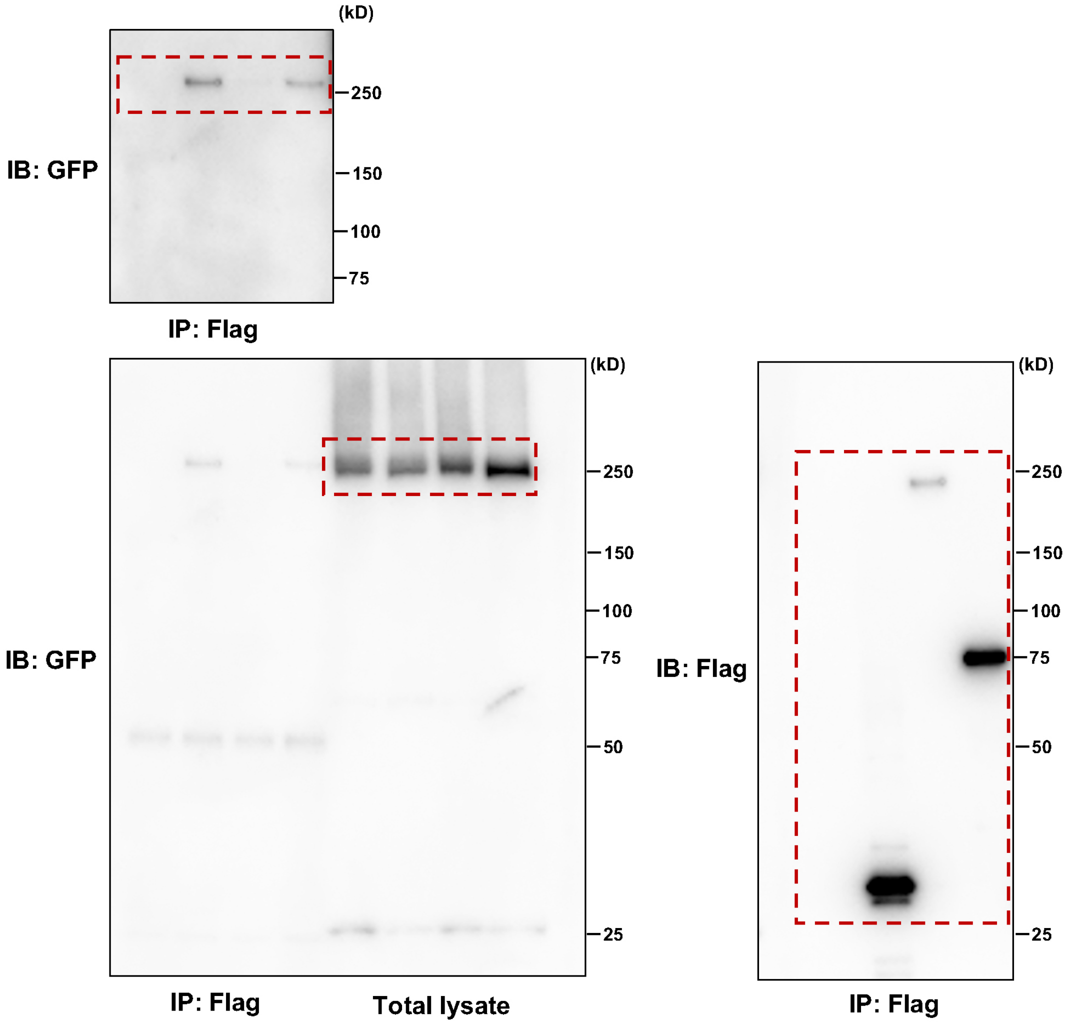

Fig. S4B

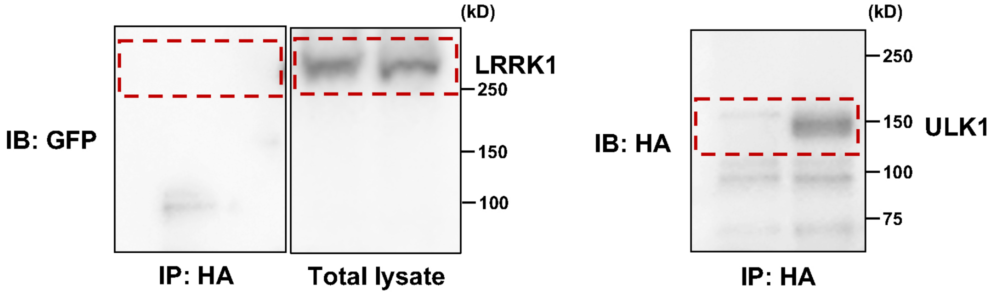

Journal of Cell Science • Supplementary information

Fig. S4C

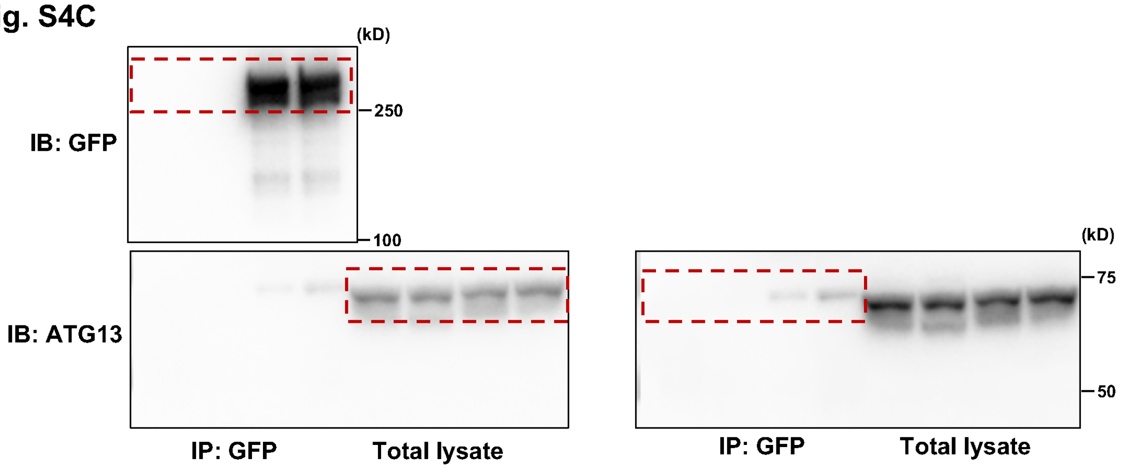

Fig. S4D

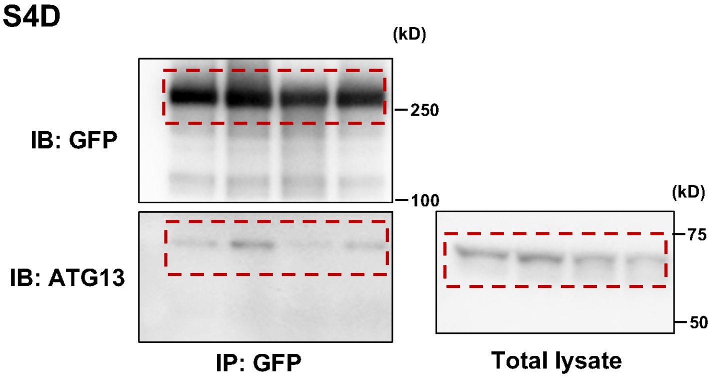

Fig. S4E

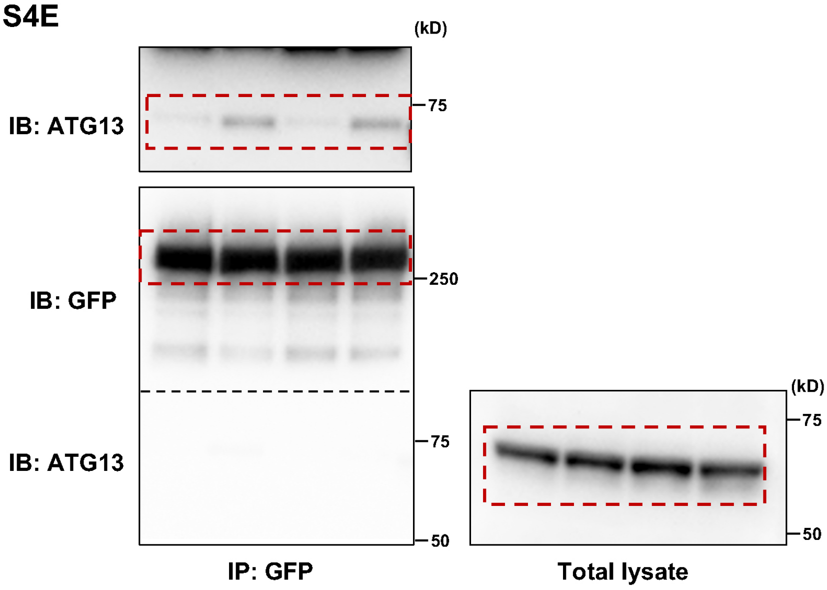

Fig. S9. Blot transparency

Journal of Cell Science • Supplementary information
