# Supplementary material for: The ULK complex–LRRK1 axis regulates Parkin-mediated mitophagy via Rab7 Ser-72 phosphorylation
Source: J Cell Sci. 2022 Dec 7;135(23):jcs260395. doi: 10.1242/jcs.260395 (PMC9789397; doi:10.1242/jcs.260395)
Supplement: Supplementary file 1 [file joces-135-260395_review_history.pdf]

The ULK complex-LRRK1 axis regulates Parkin-mediated mitophagy via Rab7 Ser-72 phosphorylation

Keitaro Fujita, Shin Kedashiro, Takuya Yagi, Naoki Hisamoto, Kunihiro Matsumoto and Hiroshi Hanafusa
DOI: 10.1242/jcs.260395

Editor: Tamotsu Yoshimori

Review timeline

Original submission:	4 July 2022
Editorial decision:	15 August 2022
First revision received:	13 October 2022
Editorial decision:	3 November 2022
Second revision received:	7 November 2022
Accepted:	16 November 2022

Original submission

First decision letter

MS ID#: JOCES/2022/260395

MS TITLE: The TBK1–ULK–LRRK1 axis regulates Parkin-mediated mitophagy via Rab7 Ser-72 phosphorylation

AUTHORS: Keitaro Fujita, Shin Kedashiro, Takuya Yagi, Naoki Hisamoto, Kunihiro Matsumoto, and Hiroshi Hanafusa
ARTICLE TYPE: Research Article

We have now reached a decision on the above manuscript.

To see the reviewers' reports and a copy of this decision letter, please go to: https://submit-jcs.biologists.org and click on the 'Manuscripts with Decisions' queue in the Author Area. (Corresponding author only has access to reviews.)

As you will see, the reviewers raise a number of substantial criticisms that prevent me from accepting the paper at this stage. They suggest, however, that a revised version might prove acceptable, if you can address their concerns. If you think that you can deal satisfactorily with the criticisms on revision, I would be pleased to see a revised manuscript. We would then return it to the reviewers.

Please ensure that you clearly highlight all changes made in the revised manuscript. Please avoid using 'Tracked changes' in Word files as these are lost in PDF conversion.

I should be grateful if you would also provide a point-by-point response detailing how you have dealt with the points raised by the reviewers in the 'Response to Reviewers' box. Please attend to all of the reviewers' comments. If you do not agree with any of their criticisms or suggestions please explain clearly why this is so.

© 2022. Published by The Company of Biologists under the terms of the Creative Commons Attribution License (https://creativecommons.org/licenses/by/4.0/).

Reviewer 1

Advance summary and potential significance to field

In this manuscript, the authors showed that LRRK1 functions downstream of ULK1/ULK2 in Parkin-mediated mitophagy. Furthermore, they showed that ectopic targeting of active LRRK1 to mitochondria is sufficient to induce the Ser-72 phosphorylation of Rab7, bypassing the requirement for TBK1 and ULK complex component ATG13. These results suggest that ULK complex recruits LRRK1 to mitochondria via its interaction with ATG13 to initiate mitophagosome formation. The authors stated that these data highlight a crucial role played by the TBK1–ULK–LRRK1 axis in the regulation of Parkin-mediated mitophagy.

This work is potentially interesting. Nevertheless, the authors should solve several experimental issues. The manuscript will be deemed suitable for acceptance upon addressing the following points.

Comments for the author

- If you would encourage revision, please detail the issues that must first be addressed. Please limit these to essential revisions, rather than potential extensions of the study.

The authors should address following weak points.

(1)
Fig. 1, Fig. S2, Fig. S5, Fig. S7
Experimental methods to monitor Mitophagy

To monitor mitophagy, the authors used immunocytochemical analysis.
Immunostaining essentially provides qualitative data.
Of course, the authors already understood the problem, and thus showed bar graphs as quantitative data.

However, mitophagy does not progress like a digital signal as “0 or 1”. For example, LRRK1 knock-down cells, the proportion of mitochondria-free cells decreased from 34% to 11%. However, the actual mitochondrial content is not digital signal (as 0 or 1), and there should be a diversity in mitochondrial content at the cellular level (such that 0, 0.3, 0.5, 0.7 or 1 mitochondria are contained per cell).

Such the analogue cell populations were divided into two groups as 'cells with Mt' and 'cells without Mt', and their numbers were counted. Consequently, quantitative accuracy seems to be lost.

To monitor mitophagy quantitatively, mito-Keima or mito-QC should be used.
Honestly speaking, I would like to ask the authors to perform such experiments.
However, given that the authors are not autophagy/mitophagy researchers, and they may not routinely measure mito-QC or mito-Keima, so the experimental hurdles may be high.

As an alternative plan, At the very least, the authors should perform immunoblotting against mitochondrial matrix protein and show that the signal decreases upon mitophagy progression.
Such immunoblotting data would provide quantitative information on mitophagy.

Of course, it might be difficult to quantify mitophagy by immunoblotting even if siRNA/plasmid transfection efficiency was very low. However, Fig. 1B suggests that the proportion of mitochondria-positive cells was reduced from 100% to 70% upon CCCP treatment and is restored to 90% by LRRK1 knock down. If so, we could expect that mitochondrial matrix protein should be reduced similarly by CCCP treatment and should be recovered similarly by LRRK1 knock-down.

(2)
Weak points in quantitative analysis of mitochondrial localization of phosphorylated Rab7.

© 2022. Published by The Company of Biologists under the terms of the Creative Commons Attribution License (https://creativecommons.org/licenses/by/4.0/).

Regarding data using GFP-LRRK1(Y944F) (Figs. 2C and 2D, Figs. 3C and 3D, and Figs. 4G and 4H), phosphorylation of S72 in Rab7 occurs in the cells, and subcellular localization of phospho-Rab7 is informative.

On the other hand, I surmise that the correct interpretations of the data shown in Figs. 1C, 1D, 1E and 1F are not 'phosphorylated Rab7 did not localize on mitochondria' but 'Rab7 was not phosphorylated'.

For Figs. 1C, 1D, 2A and 3A, where the mitochondrial localization of Rab7 phosphorylation is analyzed in immunocytochemistry, the authors should also analyze the amount of phospho-Rab7 in immunoblotting.

At the time, Phos-tag analysis of Rab7 should also be performed, since the specificity of the pRab7 antibody cannot be verified by the immunoblotting, and it is not known what amount of Rab7 is phosphorylated.

(3)
Regarding quantitative analysis of mitochondrial localization of phosphorylated Rab7, what does 'pRab7 mitochondria localisation (%)' mean?

It does not seem clear whether it is the percentage of cells with pRab7 mitochondrial localization per pRab7-positive cells, or the percentage of cells with pRab7 mitochondrial localization per total cells.

Readers might be confused by this point, ans the author should explain this point carefully.

(4)
The authors mentioned, in the text, that TBK1 is crucial for the translocation of LRRK1 to damaged mitochondria (page 11). However, I think this statement is not supported by experimental data.

Indeed, even when GFP-LRRK1 complemented mitochondrial localization of pS72 Rab7 in LRRK1 knock-down cells, mitochondrial localization of LRRK1 was not observed (Fig. 1E). If not over-expressed, did LRRK1 translocate on mitochondria upon mitophagy induction? The authors should also address this point.

Reviewer 2

Advance summary and potential significance to field

In this study, Fujita et al. report that the protein kinase LRRK1 is involved in the regulation of Parkin-mediated mitophagy in mammalian cells. In a previous study, the authors found that LRRK1 phosphorylates Ser72 of Rab7 to regulate the trafficking of EGFR, while other groups reported that Rab7 and its phosphorylation are important for the recruitment of Atg9 and LC3 to damaged mitochondria during mitophagy. In the present study, the authors show that LRRK1 phosphorylates Rab7 on damage mitochondria upon mitophagy induction in a manner dependent on TBK1 kinase and the ULK kinase complex. This dependency was bypassed when a constitutively active mutant of LRRK1 was engineered to be localized to mitochondria, suggesting that LRRK1 functions downstream of these kinases. The authors further showed that the mitochondrial recruitment of LRRK1 is mediated by the interaction with ATG13, a component of the ULK complex. Thus this study significantly advances our understanding of the mechanism of mitophagy regulation. However, the authors should address the following concerns to strengthen their conclusions or further clarify the mechanism.

Comments for the author

In this study, Fujita et al. report that the protein kinase LRRK1 is involved in the regulation of Parkin-mediated mitophagy in mammalian cells. In a previous study, the authors found that LRRK1 phosphorylates Ser72 of Rab7 to regulate the trafficking of EGFR, while other groups reported that Rab7 and its phosphorylation are important for the recruitment of Atg9 and LC3 to damaged

© 2022. Published by The Company of Biologists under the terms of the Creative Commons Attribution License (https://creativecommons.org/licenses/by/4.0/).

mitochondria during mitophagy. In the present study, the authors show that LRRK1 phosphorylates Rab7 on damage mitochondria upon mitophagy induction in a manner dependent on TBK1 kinase and the ULK kinase complex. This dependency was bypassed when a constitutively active mutant of LRRK1 was engineered to be localized to mitochondria, suggesting that LRRK1 functions downstream of these kinases. The authors further showed that the mitochondrial recruitment of LRRK1 is mediated by the interaction with ATG13, a component of the ULK complex. Thus this study significantly advances our understanding of the mechanism of mitophagy regulation. However, the authors should address the following concerns to strengthen their conclusions or further clarify the mechanism.

Specific comments:
1. How does CCCP stimulate the interaction of ATG13 with LRRK1? The authors should examine whether this stimulation depends on PINK1.

2. Does CCCP treatment specifically stimulate LRRK1 activity on damaged mitochondria (not on endosomes)?

3. The authors use a constitutively active mutant of LRRK1 in this study. They should mention how LRRK1 activity is regulated and discuss it in the context of mitophagy induction.

4. Fig. 1A: (i) The authors should confirm that mitochondria disappearance depends on autophagy (ATG genes) in their experimental settings. (ii) Where does Parkin localize when cells lost all mitochondria?

5. Page 11, “These results indicate that the ectopic localization of LRRK1(Y944F) to the mitochondria is sufficient to initiate de novo mitophagosome biogenesis without Parkin expression or mitochondrial dysfunction but does not eliminate mitochondria.”: This statement would be misleading. In the absence of Parkin or mitochondrial damage, mitochondrial protein ubiquitylation does not occur, and ubiquitin-binding adaptors including NDP52 and the ULK complex are not recruited. Therefore, autophagosome biogenesis would not be normally initiated although the recruitment of ATG9 and LC3 was observed.

6. Fig. 2C: The authors showed that LRRK1 can be recruited to mitochondria even in ULK1/2 knockdown cells when constitutive active LRRK1 was expressed. The authors should check if ATG13 accumulate on mitochondria in these cells, since they propose that ATG13 mediates the mitochondrial recruitment of LRRK1. These experiments also raised the important question how ULK is involved in LRRK1 recruitment to mitochondria and the possibility that ULK upregulates LRRK1 kinase activity (via LRRK1 phosphorylation). It would be better if the authors could address this issue.

7. Fig. 5A-D: (i) The authors showed that constitutively active LRRK1 localizes to mitochondria depending on forcible mitochondrial targeting of ATG13. The authors should examine whether wild-type LRRK1 also localizes to mitochondria in the same setting. If not, LRRK1 activation is likely to be a prerequisite for its mitochondrial localization, an important insight into the mechanism of LRRK1 recruitment to mitochondria. (ii) The authors should also examine the effect of CCCP treatment on the recruitment of LRRK1 and the levels of pS72 Rab7 with and without expressing LRRK1 Y944F.

8. Fig. 5E: The authors should examine whether the ATG13-LRRK1 interaction increases in the Y944F mutant and whether it is further increased by CCCP treatment. The results will also provide important insights into the regulation of the mitochondrial recruitment of LRRK1 upon mitochondrial damage.

© 2022. Published by The Company of Biologists under the terms of the Creative Commons Attribution License (https://creativecommons.org/licenses/by/4.0/).

First revision

Author response to reviewers' comments

Our responses to the comments of Reviewer #1

(1)
Fig. 1, Fig. S2, Fig. S5, Fig. S7
Experimental methods to monitor Mitophagy
To monitor mitophagy, the authors used immunocytochemical analysis. Immunostaining essentially provides qualitative data. Of course, the authors already understood the problem, and thus showed bar graphs as quantitative data. However, mitophagy does not progress like a digital signal as "0 or 1". For example, LRRK1 knock-down cells, the proportion of mitochondria-free cells decreased from 34% to 11%.
However, the actual mitochondrial content is not digital signal (as 0 or 1), and there should be a diversity in mitochondrial content at the cellular level (such that 0, 0.3, 0.5, 0.7 or 1 mitochondria are contained per cell). Such the analogue cell populations were divided into two groups as 'cells with Mt' and 'cells without Mt', and their numbers were counted. Consequently, quantitative accuracy seems to be lost.

We quantified the distribution of mitochondrial content based on the intensity of immunostaining for C-III core 1, a mitochondrial matrix protein (Fig. S1A, B, D, S6B, S7B).

We added the above information to the "MATERIALS AND METHODS" section (p. 20, lines 16-25).

To monitor mitophagy quantitatively, mito-Keima or mito-QC should be used. Honestly speaking, I would like to ask the authors to perform such experiments. However, given that the authors are not autophagy/mitophagy researchers, and they may not routinely measure mito-QC or mito-Keima, so the experimental hurdles may be high.

We attempted mito-Keima. However, we could not measure it.

As an alternative plan, At the very least, the authors should perform immunoblotting against mitochondrial matrix protein and show that the signal decreases upon mitophagy progression. Such immunoblotting data would provide quantitative information on mitophagy.
Of course, it might be difficult to quantify mitophagy by immunoblotting even if siRNA/plasmid transfection efficiency was very low. However, Fig. 1B suggests that the proportion of mitochondria-positive cells was reduced from 100% to 70% upon CCCP treatment and is restored to 90% by LRRK1 knock down. If so, we could expect that mitochondrial matrix protein should be reduced similarly by CCCP treatment and should be recovered similarly by LRRK1 knock-down.

We attempted to quantify mitophagy by immunoblotting against mitochondrial matrix protein C-III core 1. However, no significant differences were obtained. This failure may be due to the low transfection efficiency of Parkin plasmid, as noted by this reviewer.

(2)
Weak points in quantitative analysis of mitochondrial localization of phosphorylated Rab7. Regarding data using GFP-LRRK1(Y944F) (Figs. 2C and 2D, Figs. 3C and 3D, and Figs. 4G and 4H), phosphorylation of S72 in Rab7 occurs in the cells, and subcellular localization of phospho-Rab7 is informative. On the other hand, I surmise that the correct interpretations of the data shown in Figs. 1C, 1D, 1E and 1F are not 'phosphorylated Rab7 did not localize on mitochondria' but 'Rab7 was not phosphorylated'.

We have corrected the description based on the comment.

For Figs. 1C, 1D, 2A and 3A, where the mitochondrial localization of Rab7 phosphorylation is analyzed in immunocytochemistry, the authors should also analyze the amount of phospho-Rab7 in immunoblotting.

© 2022. Published by The Company of Biologists under the terms of the Creative Commons Attribution License (https://creativecommons.org/licenses/by/4.0/).

At the time, Phos-tag analysis of Rab7 should also be performed, since the specificity of the pRab7 antibody cannot be verified by the immunoblotting, and it is not known what amount of Rab7 is phosphorylated.

We attempted to analyze the amount of endogenous phospho-Rab7 by immunoblotting. However, as demonstrated previously (Hanafusa et al., J. Cell Sci., 2019), we could not detect the phosphorylation of endogenous Rab7 by Western blot analysis using the pS72-Rab7 antibody.

Furthermore, we could not detect endogenous phosphor-Rab7 by Phos-tag analysis. This failure may be due to the low transfection efficiency of Parkin plasmid, as noted by this reviewer.

We have already demonstrated the specificity of the anti-pS72-Rab7 antibody (Hanafusa et al., J. Cell Sci., 2019).

(3)
Regarding quantitative analysis of mitochondrial localization of phosphorylated Rab7, what does 'pRab7 mitochondria localisation (%)' mean?
It does not seem clear whether it is the percentage of cells with pRab7 mitochondrial localization per pRab7-positive cells, or the percentage of cells with pRab7 mitochondrial localization per total cells.
Readers might be confused by this point, and the author should explain this point carefully.

We have noted in the figure legend that pRab7 mitochondria localization (%) refers to the percentage of cells with pRab7 signals on structures decorated with Parkin per total Parkin-expressing cells.

(4)
The authors mentioned, in the text, that TBK1 is crucial for the translocation of LRRK1 to damaged mitochondria (page 11). However, I think this statement is not supported by experimental data.

We have corrected this description and modified the TBK1 section.

Indeed, even when GFP-LRRK1 complemented mitochondrial localization of pS72 Rab7 in LRRK1 knock-down cells, mitochondrial localization of LRRK1 was not observed (Fig. 1E). If not over-expressed, did LRRK1 translocate on mitochondria upon mitophagy induction? The authors should also address this point.

We could not detect the translocation of endogenous LRRK1 on mitochondria upon mitophagy induction.

Furthermore, the localization of overexpressed LRRK1(Y944F) on endosomes was detectable compared with that of LRRK1(Y944F) on the mitochondria upon CCCP treatment (Fig. 2C). Thus, we could not detect the translocation of LRRK1 to the mitochondria upon mitophagy activation in U2OS cells. However, we obtained the following results, suggesting that ATG13 mediates the recruitment of LRRK1 to the mitochondria.

When LRRK1(Y944F) was expressed in cells expressing mitochondria-targeted ATG13, pS72-Rab7 signals were induced on the mitochondria. Under these conditions, LRRK1(Y944) was localized to the mitochondria decorated with ATG13 (Fig. 6C).

<u>Our responses to the comments of Reviewer #2</u>

Specific comments:
1. How does CCCP stimulate the interaction of ATG13 with LRRK1? The authors should examine whether this stimulation depends on PINK1.

© 2022. Published by The Company of Biologists under the terms of the Creative Commons Attribution License (https://creativecommons.org/licenses/by/4.0/).

We have examined the effect of PINK1 depletion on the CCCP-dependent interaction of ATG13 and LRRK1. We showed that PINK1 siRNA suppressed the interaction between ATG13 and LRRK1 induced by CCCP stimulation (Fig. S3D).

2. Does CCCP treatment specifically stimulate LRRK1 activity on damaged mitochondria (not on endosomes)?

The expression of LRRK1(Y944F) induced pRab7 on endosomes (Fig. 2C), but CCCP treatment did not induce pRab7 on endosomes (Fig. 1C, E). Thus, CCCP treatment appears to specifically stimulate LRRK1 activity on damaged mitochondria.

3. The authors use a constitutively active mutant of LRRK1 in this study. They should mention how LRRK1 activity is regulated and discuss it in the context of mitophagy induction.

We showed that ULK1 phosphorylates LRRK1 (Fig. S5) and discussed the mechanism through which LRRK1 activity is regulated (p. 14, lines 25-29 and p. 15, lines 1-13).

4. Fig. 1A: (i) The authors should confirm that mitochondria disappearance depends on autophagy (ATG genes) in their experimental settings. (ii) Where does Parkin localize when cells lost all mitochondria?

We showed that mitochondria disappearance depends on ATG101 and ULK1/2 (ATG1) in our experimental settings (Fig. S6A-C, S7A-C).

When cells lost all mitochondria, Parkin is uniformly localized in the cytoplasm.

5. Page 11, "These results indicate that the ectopic localization of LRRK1(Y944F) to the mitochondria is sufficient to initiate de novo mitophagosome biogenesis without Parkin expression or mitochondrial dysfunction but does not eliminate mitochondria.": This statement would be misleading. In the absence of Parkin or mitochondrial damage, mitochondrial protein ubiquitylation does not occur, and ubiquitin-binding adaptors including NDP52 and the ULK complex are not recruited. Therefore, autophagosome biogenesis would not be normally initiated although the recruitment of ATG9 and LC3 was observed.

We have corrected this description.

6. Fig. 2C: The authors showed that LRRK1 can be recruited to mitochondria even in ULK1/2 knockdown cells when constitutive active LRRK1 was expressed. The authors should check if ATG13 accumulate on mitochondria in these cells, since they propose that ATG13 mediates the mitochondrial recruitment of LRRK1. These experiments also raised the important question how ULK is involved in LRRK1 recruitment to mitochondria and the possibility that ULK upregulates LRRK1 kinase activity (via LRRK1 phosphorylation). It would be better if the authors could address this issue.

We were unable to detect mitochondrial localization of ATG13 in U2OS cells in response to CCCP treatment.

We showed that ULK1 phosphorylates LRRK1 (Fig. S5).

We discussed the mechanism through which ULK regulates LRRK1 kinase activity. Mitophagy activation induces the recruitment of the ULK complex and LRRK1 to damaged mitochondria, resulting in the activation of ULK kinase, thereby phosphorylating and activating LRRK1.

7. Fig. 5A-D: (i) The authors showed that constitutively active LRRK1 localizes to mitochondria depending on forcible mitochondrial targeting of ATG13. The authors should examine whether wild-type LRRK1 also localizes to mitochondria in the same setting. If not, LRRK1 activation is likely to be a prerequisite for its mitochondrial localization, an important insight into the mechanism of LRRK1 recruitment to mitochondria. (ii) The authors should also examine the effect of CCCP treatment on the recruitment of LRRK1 and the levels of pS72 Rab7 with and without expressing LRRK1 Y944F.

© 2022. Published by The Company of Biologists under the terms of the Creative Commons Attribution License (https://creativecommons.org/licenses/by/4.0/).

We examined whether wild-type LRRK1 also localizes to the mitochondria in the same setting. We showed that the co-expression of wild-type Myc-LRRK1 in cells expressing FKBP-GFP-ATG13 and FRB-Fis1 induced mitochondrial localization of wild-type LRRK1 upon the addition of rapalog but not Rab7 Ser-72 phosphorylation on mitochondria (Fig. 4E, F). These results suggest that ATG13 has two functions concerning LRRK1 in Parkin-mediated mitophagy: the recruitment of LRRK1 to damaged mitochondria and its role in mediating LRRK1 activation.

8. Fig. 5E: The authors should examine whether the ATG13-LRRK1 interaction increases in the Y944F mutant and whether it is further increased by CCCP treatment. The results will also provide important insights into the regulation of the mitochondrial recruitment of LRRK1 upon mitochondrial damage.

We showed that the interaction of overexpressed LRRK1 (Y944F) with ATG13 was also dependent on the CCCP stimulation (Fig. S3E).

Second decision letter

MS ID#: JOCES/2022/260395

MS TITLE: The ULK complex–LRRK1 axis regulates Parkin-mediated mitophagy via Rab7 Ser-72 phosphorylation

AUTHORS: Keitaro Fujita, Shin Kedashiro, Takuya Yagi, Naoki Hisamoto, Kunihiro Matsumoto, and Hiroshi Hanafusa
ARTICLE TYPE: Research Article

We have now reached a decision on the above manuscript.

To see the reviewers' reports and a copy of this decision letter, please go to: https://submit-jcs.biologists.org and click on the 'Manuscripts with Decisions' queue in the Author Area. (Corresponding author only has access to reviews.)

As you will see, the reviewers gave favourable reports but raised some critical points that will require amendments to your manuscript. I hope that you will be able to carry these out because I would like to be able to accept your paper, depending on further comments from reviewers.

Please ensure that you clearly highlight all changes made in the revised manuscript. Please avoid using 'Tracked changes' in Word files as these are lost in PDF conversion.

I should be grateful if you would also provide a point-by-point response detailing how you have dealt with the points raised by the reviewers in the 'Response to Reviewers' box. Please attend to all of the reviewers' comments. If you do not agree with any of their criticisms or suggestions please explain clearly why this is so.

Reviewer 1

Advance summary and potential significance to field

The outline of this manuscript is that LRRK1 positively regulates mitophagy by phosphorylating RAB7A Ser72. The manuscript also reports that LRRK1 binds to ATG13, which is a point of contact between LRRK1 and mitophagy.

Comments for the author

The main problem with the original manuscript is that mitochondrial degradation was only observed as immune-fluorescence data. I pointed this out in my first review comments.

© 2022. Published by The Company of Biologists under the terms of the Creative Commons Attribution License (https://creativecommons.org/licenses/by/4.0/).

Of course, even in the original manuscript, the authors showed data analyzing multiple cells as Fig. 1B. However, during such analysis, individual cell was converted to a digital signal (0 or 1) depending on the presence or absence of mitochondria. As the actual mitochondrial abundance is continuous, we do not fully agree that quantitative analysis is performed by increasing the N of such digitized data.

Originally, experiments using mitophagy monitoring probes such as mito-Keima and mito-QC should be performed to measure mitophagy. On the other hand, given that the authors are not experts in autophagy research, immunoblotting analysis against mitochondrial matrix protein should be performed. These points were described in the first review comments.

This time, through Revision process, neither mito-Keima analysis nor immune-blotting against matrix protein were presented. The authors stated that the low efficiency of Parkin transfection is the reason for the difficulty to perform such analysis. However, this can be solved by using Parkin stably expressing cell lines.

On the other hand, the authors stated that "We quantified the distribution of mitochondrial content based on the intensity of immunostaining for C-III core 1, a mitochondrial matrix protein (Fig. S1A, B, D, S6B, S7B)". Thus, we can realize that the authors tried to grasp mitochondrial abundance as continuous values.

Given that data such as Fig. S1 are more credible than data such as Fig. 1B, and that the data in this manuscript will be the standards for the publication of papers regarding mitophagy in JCS, newly added quantitative data on mitochondria such as Fig. S1 should be shown in the main Figure.

If this is not possible due to space limitations, the authors should replace digitized cellular data such as Fig. 1B with quantitative data such as Figs. S1A-B. Only after making such changes, the manuscript becomes suitable for acceptance.

Reviewer 2

Advance summary and potential significance to field

The authors have satisfactorily addressed the issues I raised for the original manuscript. Given the additional result showing that the CCCP-dependent ATG13-LRRK1 interaction requires PINK1, PINK1 should be included in the model shown in Fig. 7.

Comments for the author

The authors have satisfactorily addressed the issues I raised for the original manuscript. Given the additional result showing that the CCCP-dependent ATG13-LRRK1 interaction requires PINK1, PINK1 should be included in the model shown in Fig. 7.

Second revision

Author response to reviewers' comments

Our responses to the comments of Reviewer #1

Given that data such as Fig. S1 are more credible than data such as Fig. 1B, and that the data in this manuscript will be the standards for the publication of papers regarding mitophagy in JCS, newly added quantitative data on mitochondria such as Fig. S1 should be shown in the main Figure.

© 2022. Published by The Company of Biologists under the terms of the Creative Commons Attribution License (https://creativecommons.org/licenses/by/4.0/).

If this is not possible due to space limitations, the authors should replace digitized cellular data such as Fig. 1B with quantitative data such as Figs. S1A-B. Only after making such changes, the manuscript becomes suitable for acceptance.

According to the reviewer's suggestion, we have shown quantitative data on mitochondria in the main Figure (new Fig. 1B).

<u>Our responses to the comments of Reviewer #2</u>

The authors have satisfactorily addressed the issues I raised for the original manuscript. Given the additional result showing that the CCCP-dependent ATG13-LRRK1 interaction requires PINK1, PINK1 should be included in the model shown in Fig. 7.

According to the reviewer's suggestion, we have included PINK1 in the model shown in new Fig. 8.

<u>Third decision letter</u>

MS ID#: JOCES/2022/260395

MS TITLE: The ULK complex-LRRK1 axis regulates Parkin-mediated mitophagy via Rab7 Ser-72 phosphorylation

AUTHORS: Keitaro Fujita, Shin Kedashiro, Takuya Yagi, Naoki Hisamoto, Kunihiro Matsumoto, and Hiroshi Hanafusa
ARTICLE TYPE: Research Article

I am happy to tell you that your manuscript has been accepted for publication in Journal of Cell Science, pending standard ethics checks.

<u>Reviewer 1</u>

Advance summary and potential significance to field

The outline of this manuscript is that LRRK1 positively regulates mitophagy by phosphorylating RAB7A Ser72. The manuscript also reports that LRRK1 binds to ATG13, which is a point of contact between LRRK1 and mitophagy.

Comments for the author

The authors have satisfactorily addressed the issues I raised for the 1st-round revision manuscript. Thus the 2nd-round revision manuscript became suitable for acceptance now.

<u>Reviewer 2</u>

Advance summary and potential significance to field

I think the revised manuscript is now suitable for publication in Journal of Cell Science.

Comments for the author

I think the revised manuscript is now suitable for publication in Journal of Cell Science.

© 2022. Published by The Company of Biologists under the terms of the Creative Commons Attribution License (https://creativecommons.org/licenses/by/4.0/).
